# The Si_2_H radical supported by two N-heterocyclic carbenes†
†Electronic supplementary information (ESI) available: Cyclic voltammetric studies of **1H**[B(Ar^F^)_4_]; synthesis, analytical data and illustrations of the IR and UV-Vis spectra of **1H**; details of the magnetic susceptibility measurements and single crystal X-ray diffraction analysis of **1H**; details of the EPR spectroscopic measurements and illustrations of the EPR spectra of **1H**; details of the quantum chemical calculations. CCDC 1471165. For ESI and crystallographic data in CIF or other electronic format see DOI: 10.1039/c6sc01569g


**DOI:** 10.1039/c6sc01569g

**Published:** 2016-05-09

**Authors:** Marius I. Arz, Gregor Schnakenburg, Andreas Meyer, Olav Schiemann, Alexander C. Filippou

**Affiliations:** a Institute of Inorganic Chemistry , University of Bonn , Gerhard-Domagk-Str. 1 , D-53121 , Bonn , Germany . Email: filippou@uni-bonn.de; b Institute of Physical and Theoretical Chemistry , University of Bonn , Wegelerstr. 12 , D-53115 , Bonn , Germany

## Abstract

Cyclic voltammetric studies of the hydridodisilicon(0,II) borate [(Idipp)(H)Si^II^=Si^0^(Idipp)][B(Ar^F^)_4_] (**1H**[B(Ar^F^)_4_], Idipp = C[N(C_6_H_3_-2,6-*i*Pr_2_)CH]_2_, Ar^F^ = C_6_H_3_-3,5-(CF_3_)_2_) reveal a reversible one-electron reduction at a low redox potential (*E*_1/2_ = –2.15 V *vs.* Fc^+^/Fc). Chemical reduction of **1H**[B(Ar^F^)_4_] with KC_8_ affords selectively the green, room-temperature stable mixed-valent disilicon(0,I) hydride Si_2_(H)(Idipp)_2_ (**1H**), in which the highly reactive Si_2_H molecule is trapped between two N-heterocyclic carbenes (NHCs). The molecular and electronic structure of **1H** was investigated by a combination of experimental and theoretical methods and reveals the presence of a π-type radical featuring a terminal bonded H atom at a flattened trigonal pyramidal coordinated Si center, that is connected *via* a Si–Si bond to a bent two-coordinated Si center carrying a lone pair of electrons. The unpaired electron occupies the Si=Si π* orbital leading to a formal Si–Si bond order of 1.5. Extensive delocalization of the spin density occurs *via* conjugation with the coplanar arranged NHC rings with the higher spin density lying on the site of the two-coordinated silicon atom.

## Abstract 2

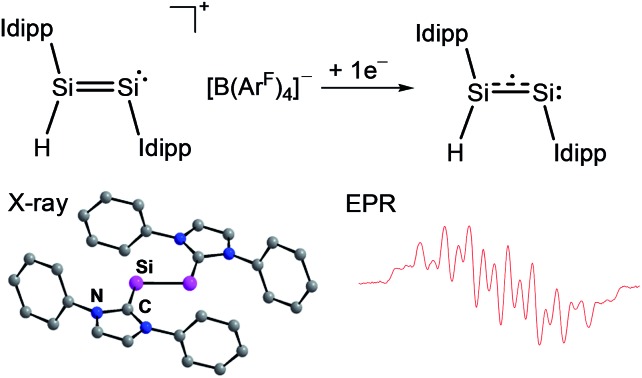
A comprehensive experimental and quantum chemical study of the open-shell mixed valent disilicon(0,I) hydride Si_2_(H)(Idipp)_2_ (Idipp = C[N(C_6_H_3_-2,6-*i*Pr_2_)CH]_2_) is reported.

## 1. Introduction

Open-shell silicon hydrides are of significant importance as transient intermediates in the chemical vapor deposition (CVD) of silicon or silicon-containing thin films, which are extensively used in the semiconductor industry.^1^ Fundamental species in the gas phase include the SiH_*x*_ (*x* = 1–3) and Si_2_H_*x*_ (*x* = 1–5) molecules as well as higher aggregated Si_*n*_H_*m*_ clusters, which are formed from silane (SiH_4_) or disilane (Si_2_H_6_) in a complex cascade of reactions.^1^ These species, which are also of interest in astrochemistry,^2^ are unstable under terrestrial conditions and can only be detected by spectroscopic or mass spectrometric techniques.^3^ One scarcely studied species in this context is the Si_2_H molecule, which was so far only detected by vibrationally-resolved photoelectron spectroscopy of Si_2_H^–^ anions.^4^ Quantum chemical calculations of Si_2_H suggest two almost isoenergetic, *C*_2v_-symmetric H-bridged structures, in which the unpaired electron occupies either the Si–Si π-bonding orbital (^2^B_1_ state) or a σ-type molecular orbital corresponding to the in-phase combination of the Si lone pair orbitals (^2^A_1_ state).^5^

Recently, N-heterocyclic carbenes (NHCs) were found to be particularly suitable Lewis bases for the thermodynamic and kinetic stabilization of highly reactive, unsaturated, low-valent Si species, leading to the isolation of a series of novel compounds with intriguing synthetic potential.^6^,^7^ Several CAAC-stabilized open-shell silicon compounds (CAAC = cyclic alkyl(amino)carbene) were also reported, in which the unpaired electron is mainly located on the CAAC substituent.^8^ Trapping of Si_2_H by NHCs appeared therefore an achievable, albeit very challenging goal, given the fact that isolable molecular hydrides of silicon in an oxidation state <2 are very rare^9^,^10^ and open-shell congeners presently unknown. In comparison, three-coordinate Si^II^ hydrides^11^ and four-coordinate Si^II^ hydrides of the general formula (LB)SiH(X)(LA) (LB = neutral Lewis base; LA = neutral Lewis acid; X = singly bonded substituent)^12^ are meanwhile well documented.

## 2. Results and discussion

**Fig. 1:**
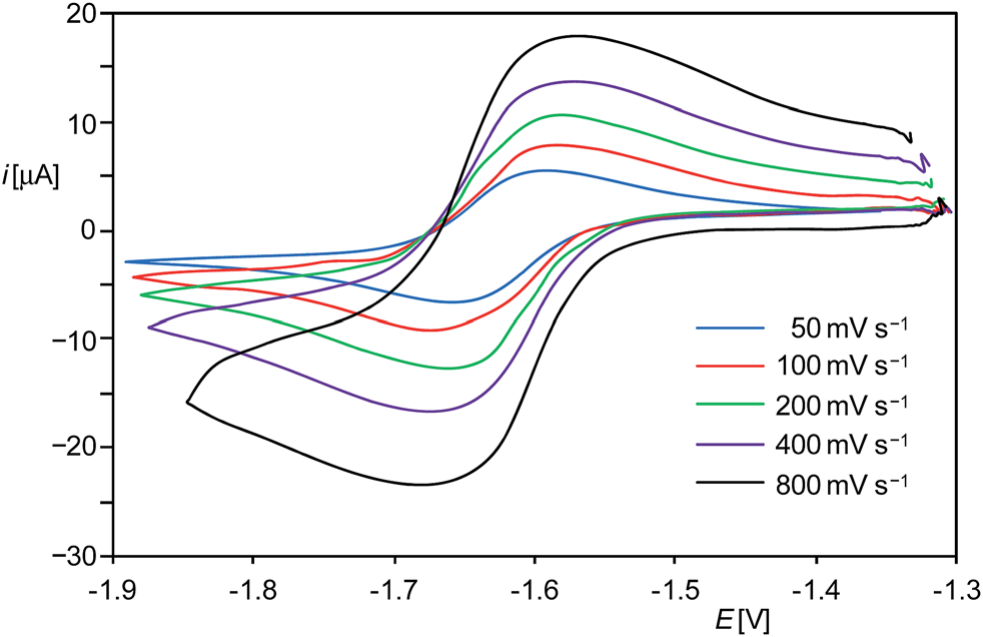
Single-scan cyclic voltammograms of **1H**[B(Ar^F^)_4_] from (–1.9) to (–1.3) V at different scan rates at room temperature in fluorobenzene/0.1 M (*n*Bu_4_N)PF_6_ solution; reference electrode: 4 mM [Fe(η^5^-C_5_Me_5_)_2_]^+1/0^/0.1 M (*n*Bu_4_N)PF_6_ in fluorobenzene.

The hydridodisilicon(0,II) salt [(Idipp)(H)Si^II^=Si^0^(Idipp)][B(Ar^F^)_4_] (**1H**[B(Ar^F^)_4_], Idipp = C[N(C_6_H_3_-2,6-*i*Pr_2_)CH]_2_, Ar^F^ = C_6_H_3_-3,5-(CF_3_)_2_), which was isolated recently in our group upon protonation of Si_2_(Idipp)_2_ (**1**),^9^ appeared to be a suitable starting material to tackle the problem of isolating an NHC-trapped Si_2_H radical. Quantum chemical studies revealed the same sequence of frontier orbitals in **1H^+^** and its isolobal phosphorus counterpart [R_2_P=PR]^+^, according to which the HOMO–1 corresponds to the lone-pair orbital at the two-coordinated E atom (E = Si, P), the HOMO is the E=E π-bonding orbital and the LUMO is the E=E π* orbital.^9^ This isolobal interrelationship suggested that **1H^+^** might be also reversibly reducible as the phosphanylphosphenium cation [Mes*(Me)P=PMes*]^+^ (Mes* = C_6_H_2_-2,4,6-*t*Bu_3_).^13^ In fact, cyclic voltammetric (CV) studies of **1H**[B(Ar^F^)_4_] in fluorobenzene at room temperature revealed a reversible one-electron reduction at a rather low half-wave potential (*E*_1/2_) of –1.63 V as well as an irreversible oxidation at +0.67 V *versus* the [Fe(η^5^-C_5_Me_5_)_2_]^+1/0^ reference electrode (Fig. 1 and ESI†).^14^ The methyl analogue [(Idipp)(Me)Si^II^=Si^0^(Idipp)][B(Ar^F^)_4_] (**1Me**[B(Ar^F^)_4_])^9^ was found also to undergo a reversible one-electron reduction, albeit at a more negative potential (*E*_1/2_ = –1.85 V) than **1H**[B(Ar^F^)_4_]. Notably, reduction of **1H^+^** and **1Me^+^** occurs at much lower potentials than that of the cation [Mes*(Me)P=PMes*]^+^ (*E*_1/2_ = –0.48 V).^13^ This marked difference in the redox potentials of the Si- and P-based cations can be rationalized with the large increase of the LUMO energy occurring upon replacement of the two PMes* fragments by the much less electronegative isolobal Si(Idipp) fragments as suggested by quantum chemical calculations.^9^

**Scheme 1:**
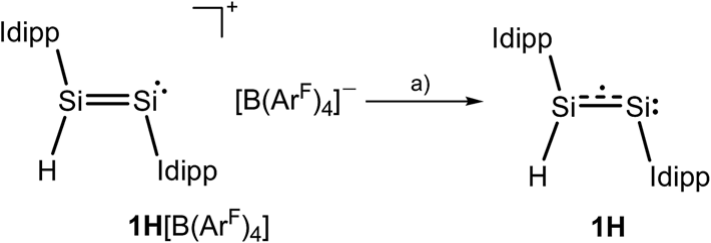
Synthesis of **1H** upon one-electron reduction of **1H**[B(Ar^F^)_4_]; (a) +KC_8_, –K[B(Ar^F^)_4_], –8C; THF; –196 °C → –40 °C. Two dots indicate a lone pair of electrons and the dotted line indicates the population of the Si=Si π* orbital upon reduction; formal charges are omitted for clarity.

The CV results prompted us to attempt a chemical one-electron reduction of **1H**[B(Ar^F^)_4_]. Indeed, vacuum transfer of THF to a 1 : 1 stoichiometric mixture of **1H**[B(Ar^F^)_4_] and KC_8_ at –196 °C followed by warming to –40 °C resulted in a distinct color change of the dark red solution of **1H**[B(Ar^F^)_4_] to give an intensely dark green solution, which after work-up and crystallization from *n*-hexane at –60 °C afforded Si_2_(H)(Idipp)_2_ (**1H**) as a dark green, almost black crystalline solid in 55% yield (Scheme 1) (see ESI†). Compound **1H** is extremely air-sensitive and immediately decolorizes upon contact with air, but can be stored under an atmosphere of argon at –30 °C without any color change or signs of decomposition in its EPR spectrum. Thermal decomposition of **1H** in a vacuum-sealed glass capillary was detected upon melting at 147 °C leading to a dark red mass. Analysis of the soluble part of the melting residue in C_6_D_6_ by ^1^H NMR spectroscopy revealed the presence of Idipp (95%) and **1** (5%).

Notably, the redox potential of **1H** [*E*_1/2_ in C_6_H_5_F = –2.15 V *vs.* [Fe(η^5^-C_5_H_5_)_2_]^+1/0^ (Fc^+^/Fc)]^15^ lies in-between that of the benzophenone radical anion (*E*_1/2_ in THF = –2.30 V *vs.* Fc^+^/Fc)^16^ and [Co(η^5^-C_5_Me_5_)_2_] (*E*_1/2_ in MeCN = –1.91 V *vs.* Fc^+^/Fc),^16^ indicating that the radical **1H** is a very strong one-electron reducing agent. Consequently, the radical **1H** is selectively oxidized back to **1H**[B(Ar^F^)_4_] upon treatment with one equivalent of [Fe(η^5^-C_5_Me_5_)_2_][B(Ar^F^)_4_] in THF-*d*_8_ (see ESI†). Thereby, the redox pair **1H^+^**/**1H** provides a very rare example of a chemically reversible Si-based redox system.7c,^17^

**Fig. 2:**
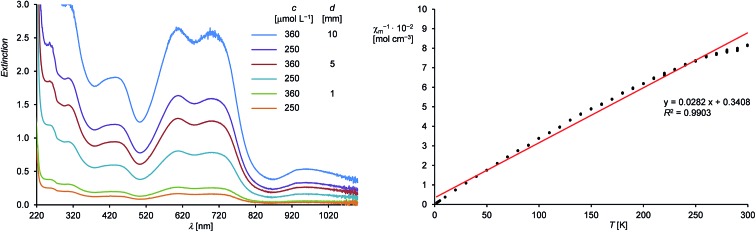
Left: UV-Vis-NIR spectra of **1H** in *n*-hexane from 220–1100 nm at different concentrations (*c*) and path lengths (*d*). Right: Plot of the reciprocal molar magnetic susceptibility (*χ*_m_^–1^) against the absolute temperature (*T*) (dotted black line) and the corresponding line (red) and line equation obtained by linear regression.

Compound **1H** is well soluble in *n*-hexane, benzene, diethyl ether or THF affording intensely dark-green solutions, even at low concentrations. The origin of this intense color was analyzed by UV-Vis-NIR spectroscopy of **1H** in *n*-hexane (Fig. 2, left and ESI†), which revealed electronic absorptions in the whole spectral range from 220–1100 nm. Six absorption maxima were located at 254 (9970), 305 (8140), 436 (5170), 608 (7110), 704 (6860) and 958 (1440) nm, of which the intense absorptions at 608 and 704 nm are responsible for the green color of **1H** (the values of the molar absorption coefficients *ε*_λ_ are given in brackets in L mol^–1^ cm^–1^). The UV-Vis-NIR spectrum was also analyzed by time-dependent density functional theory (TdDFT) calculations (see ESI, Fig. S21†).^18^

Magnetic susceptibility measurements of solid **1H** from 300.0–1.9 K suggest the presence of a paramagnetic compound with one unpaired electron following Curie's law. A plot of the reciprocal molar magnetic susceptibility (*χ*_m_^–1^) against the absolute temperature (*T*) showed a linear correlation from which the effective magnetic moment *μ*_eff_ was calculated after linear regression and found to be 1.68 *μ*_B_ (Fig. 2, right and ESI†). This value is slightly lower than the value derived from the spin-only formula for one unpaired electron (*μ*_eff_ = 1.73 *μ*_B_).

**Fig. 3:**
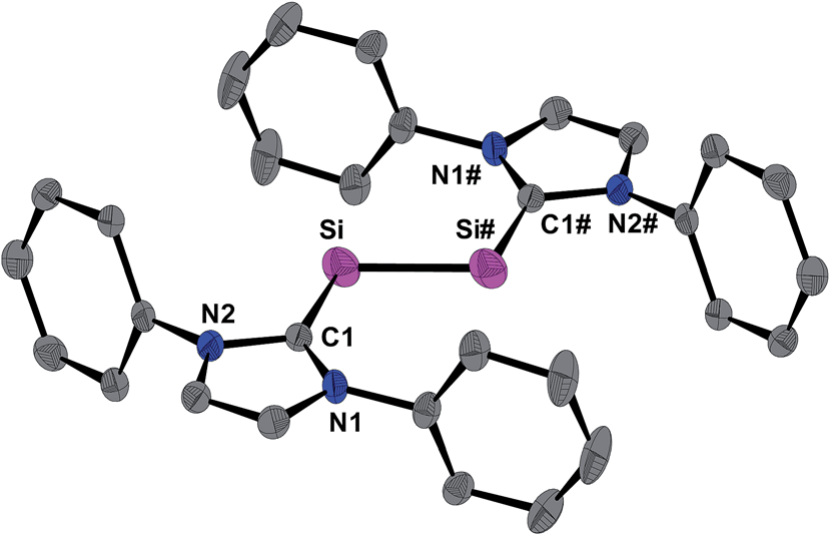
DIAMOND plot of the molecular structure of **1H** in the single crystal at 123(2) K. Thermal ellipsoids are set at 30% electronic probability. The hydrogen atoms and the *i*Pr groups are omitted for clarity. The Si-bonded H atom was omitted due to its uncertain position. Selected bond lengths [Å], bond angles [°] and torsion angles [°]: Si–Si# 2.281(3), Si–C1 1.873(4); C1–Si–Si# 109.5(1); C1–Si–Si#–C1# 180.0(3).

The molecular structure of **1H** was determined by single crystal X-ray crystallography. The radical features a crystallographically imposed inversion symmetry (space group: *P*2_1_/*c*) in marked contrast to the *C*_1_-symmetric structure of **1H^+^** in **1H**[B(Ar^F^)_4_].^9^ The Si-bonded H atom was located in the difference Fourier map and anisotropically refined with a site occupancy of 1/2 at each Si atom. However, the exact position of this H atom could not be deduced by X-ray crystallography, since structural refinements with either a terminal (Si–H) or a bridging (Si–H–Si) position led to identical w*R*_2_ values. **1H** features as **1H**[B(Ar^F^)_4_] and **1** a *trans*-bent planar C_NHC_–Si–Si–C_NHC_ core (Fig. 3). However, distinct structural differences become apparent upon comparing the three structures. For example, the Si–Si bond of **1H** (2.281(3) Å) is considerably longer than that in **1H**[B(Ar^F^)_4_] (2.1873(8) Å)^9^ or **1** (2.229(1) Å)^19^ (Table 1), and lies in-between that of a typical Si=Si double bond (2.20 Å)^20^ and a Si–Si single bond (*e.g.* 2.352 Å in α-Si).^21^ In comparison, the Si–C_NHC_ bonds in **1H** (1.873(4) Å) are shorter than the Si–C_NHC_ bonds of the dicoordinated Si atoms in **1H**[B(Ar^F^)_4_] (1.940(2) Å)^9^ and **1** (1.927(1) Å)^19^ (Table 1), and similar to that of the trigonal-planar coordinated Si atom in **1H**[B(Ar^F^)_4_] (1.882(2) Å).^9^ Reduction of **1H^+^** results also in a distinct change of the conformation of the NHC substituents. Thus, both N-heterocyclic rings in **1H** are arranged coplanar with the *trans*-bent C_NHC_–Si–Si–C_NHC_ core as evidenced by the dihedral angle *φ*_NHC_ of 3.3(2)° (Table 1), whereas in **1H^+^** one of the two N-heterocyclic rings (bonded to the two-coordinated Si atom) adopts an almost orthogonal orientation (Table 1). All these structural changes can be rationalized by quantum theory (*vide infra*). Thus, reduction of **1H^+^** leads to a population of the Si=Si π* orbital with one electron, reducing thereby the formal Si–Si bond order from 2 in **1H^+^** to 1.5 in **1H** as nicely reflected in the computed Si–Si Wiberg bond indexes (WBI; WBI(Si–Si) of **1H^+^** = 1.70; WBI(Si–Si) of **1H** = 1.17) (see ESI, Tables S11 and S12†). The coplanar arrangement of the N-heterocyclic rings allows for an optimal in-phase interaction (π-conjugation) of the Si=Si π* orbital with π*(CN_2_) orbitals of the NHC substituents in the SOMO of **1H** (Fig. 6), providing a rationale for the shortening of the Si–C_NHC_ bonds and the concomitant elongation of the C_NHC_–N_NHC_ bonds of **1H***versus***1H^+^** (Table 1).

**Table 1:** Comparison of selected bonding parameters of **1H**, **1H**[B(Ar^F^)_4_] and **1**

	Si–Si [Å]	Si–C_NHC_ [Å]	C_NHC_–N_NHC_ [Å]	C_NHC_–Si–Si [°]	*φ*_NHC_ ^*c*^ [°]
**1H**	2.281(3)	1.873(4)	1.381(4), 1.402(4)	109.5(1)	3.3(2)
**1H**[B(Ar^F^)]_4_^*a*^	2.1873(8)	1.882(2) (Si1–C_NHC_)	1.356(2), 1.358(2)	116.73(7) (C1–Si1–Si2)	8.60(6) (*φ*_NHC1_)
		1.940(2) (Si2–C_NHC_)	1.356(2), 1.358(2)	95.34(6) (C28–Si2–Si1)	71.06(6) (*φ*_NHC2_)
**1** ^*b*^	2.229(1)	1.927(2)	1.368(2), 1.372(2)	93.37(5)	87.11(5)

^*a*^Data taken from ref. 9. Connectivity: [(NHC1)(H)Si1=Si2(NHC2)]^+^.

^*b*^Data taken from ref. 19.

^*c*^ *φ*_NHC_ denotes the dihedral angles between the C_NHC_–Si–Si–C_NHC_ least-square plane and the respective N-heterocyclic ring least-square planes.

IR spectroscopy proved to be a very useful method to determine unequivocally the position of the Si-bonded H atom. In fact, the ATR FT-IR spectrum of **1H** displayed a *ν*(Si–H) absorption band at 2089 cm^–1^, which is characteristic for stretching vibrations of terminal Si–H bonds (see ESI, Fig. S4†). In comparison, the *ν*(Si–H–Si) band of Si_2_H is predicted at significantly lower wavenumbers (1592 cm^–1^ (^2^A_1_ state); 1491 cm^–1^ (^2^B_1_ state)),^4^ and also the *ν*(Si–H–Si) absorption bands of H-bridged silylium ions are shifted to much lower wavenumbers (*ca.* 1750–1950 cm^–1^; *e.g.* 1900 cm^–1^ in [Et_3_Si–H–SiEt_3_][CHB_11_Cl_11_]) compared with the *ν*(Si–H) bands of the corresponding silanes (*ca.* 2150 cm^–1^).^22^ Notably, the *ν*(Si–H) absorption band of **1H** appears in-between that of **1H**[B(Ar^F^)_4_] containing a trigonal planar coordinated Si atom (*ν*(Si–H) = 2142 cm^–1^),^9^ and the Si(ii)-hydride (IMe_4_)SiH(Si*t*Bu_3_) containing a strongly pyramidal bonded Si atom (IMe_4_ = C[N(Me)CMe]_2_: *ν*(Si–H) in KBr = 1984 cm^–1^).11*d* Apparently, the *ν*(Si–H) frequency decreases with increasing pyramidalization of the Si atom, which according to the quantum chemical calculations can be traced back to the decreasing s-character of the Si hybrid orbital in the Si–H bond (see ESI, Tables S11 and S12†).

**Fig. 4:**
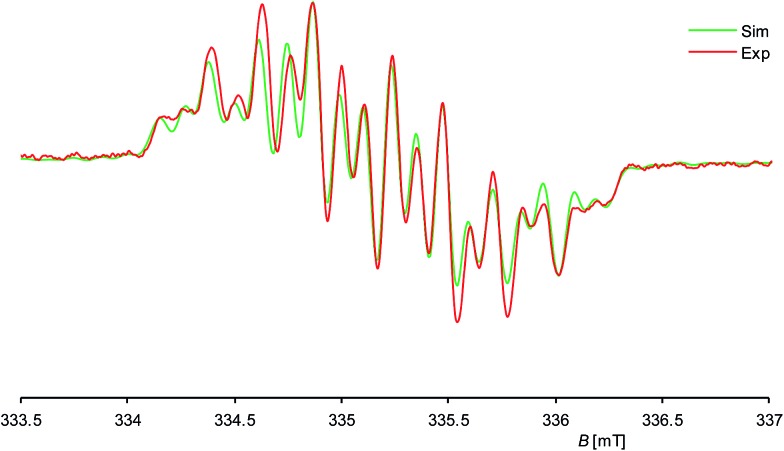
Experimental (red curve) and simulated (green curve) X-band EPR spectra of **1H** in *n*-hexane at 336 K; the ordinate (d*A*/d*B*) is omitted for clarity. *g*_iso_ = 2.00562, *a*(^29^Si1) = 1.725 mT, *a*(^29^Si2) = 0.431 mT, *a*(^14^N1) = 0.246 mT, *a*(^14^N2) = 0.100 mT, *a*(^1^H) = 0.605 mT.

Further insight into the structure of **1H** was provided by continuous wave (cw) EPR spectroscopy at X-band frequencies. Spectra with a nicely resolved hyperfine coupling pattern could be obtained from samples of **1H** in *n*-hexane solution at 336 K (Fig. 4; see also ESI, Fig. S10† for EPR spectra at different temperatures). Notably, a similar EPR spectrum was obtained in diethyl ether solution at 298 K (see ESI, Fig. S12†), suggesting that solvent coordination effects are negligible. The EPR spectrum of **1H** displays a multiplet at a *g*_iso_ value of 2.00562, which could be well simulated assuming coupling of the unpaired electron to one ^1^H (*I* = 1/2) nucleus, two different ^29^Si (*I* = 1/2) and two pairs of two magnetically equivalent ^14^N (*I* = 1) nuclei, respectively (Fig. 4). These observations suggest that **1H** has a rigid structure and does not undergo a reversible 1,2-H-migration in solution in contrast to **1H^+^**.^9^ Remarkably, two quite different *a*(^29^Si) hyperfine coupling constants (1.725 and 0.431 mT) were found, indicating an asymmetric spin density distribution over the Si atoms. Both values are smaller than those of other Si-based π type radicals, such as the disilene radical cation [Si_2_(Si*t*Bu_2_Me)_4_]^+^ (2.30 mT)^23^ or the disilene radical anions [Si_2_R_4_]^–^ (2.45–4.83 mT, R = silyl substituent)^24^ due to extensive delocalization of the spin density into the NHC substituents, and also significantly smaller than that of the σ-type radical cation in **1**[B(Ar^F^)_4_] (5.99 mT),7*c* indicating a localization of the unpaired electron in a molecular orbital of π-symmetry in agreement with the results of the quantum chemical calculations (*vide infra*). The two *a*(^14^N) hfcc's (0.246 and 0.100 mT) suggest a fast rotation of the magnetically different NHC substituents about the Si–C_NHC_ bonds on the EPR timescale occurring even at low temperature (see ESI, Table S6†).

**Fig. 5:**
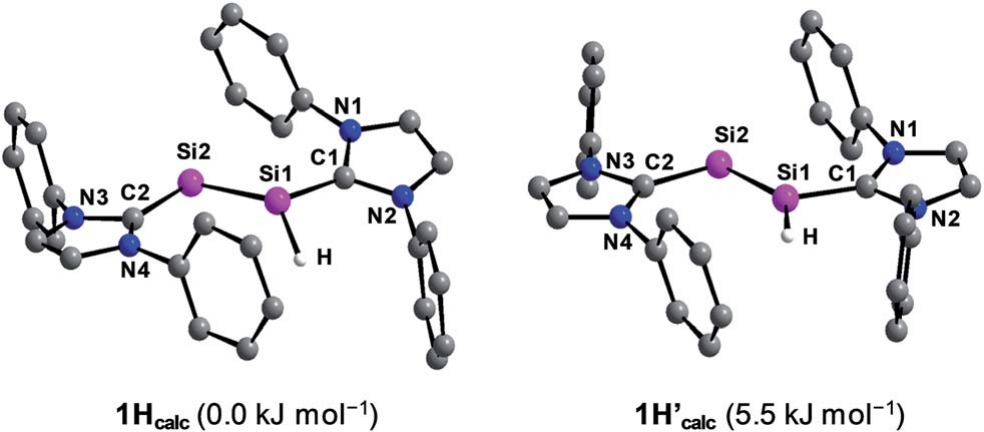
Calculated minimum structures (**1H_calc_** and **1H′_calc_**) of Si_2_(H)(Idipp)_2_ at the B97-D3/RI-JCOSX/def2-TZVP level of theory. The relative energies are given in brackets. The H atoms, except the H atom bonded to Si1, and the *i*Pr substituents are omitted for clarity.

Quantum chemical calculations of **1H** were carried out using the B3LYP functional in combination with the 6-311G** basis set for the Si, N, Si-bonded H and NHC ring C atoms and the 6-31G* basis set for the peripheral C and H atoms or the B97-D3 functional in combination with RI-JCOSX approximations and the def2-TZVP basis set for all atoms.^25^ The levels of theory are abbreviated in the following with B3LYP/I and B97-D3/II. Remarkably, calculations at the B3LYP/I level of theory yielded one minimum structure (**1H_calc_**), whereas two almost degenerate minimum structures were obtained at the B97-D3/II level of theory (**1H_calc_** and **1H′_calc_**) (Fig. 5). All calculated minimum structures display a terminally bonded H atom bound to the Si1 atom. No minimum structure with a bridged H atom was found on the potential energy hypersurface of **1H** at both levels of theory. The geometrical parameters of the minimum structure calculated at the B3LYP/I level of theory and the global minimum structure at the B97-D3/II level of theory are almost identical (Table 2 and ESI, Table S8†). These structures (**1H_calc_**) contain a trigonal-pyramidal coordinated Si1 atom with a sum of angles of 335.51° (B3LYP/I) and 342.58° (B97-D3/II), respectively. Remarkably, the calculated structure of the diphosphanyl radical P_2_(Me)Mes*2, which is isolobal to **1H**, displays a trigonal pyramidal geometry at the three-coordinated P atom (sum of angles: 337.5°),^13^ as found for **1H_calc_**. In comparison, the second minimum structure obtained at the B97-D3/II level of theory (**1H′_calc_**) is only 5.5 kJ mol^–1^ higher in energy than **1H_calc_** and contains the Si1 atom in a trigonal planar environment (sum of angles: 359.61°). A comparison of the structural parameters of **1H_calc_** and **1H′_calc_** with those obtained by single crystal X-ray diffraction reveals a good agreement of the calculated Si–Si, Si–C_NHC_ and C_NHC_–N_NHC_ bond lengths of both minimum structures (Table 2 and ESI, Table S8†). While the experimental results did not allow to clearly distinguish whether a flattened trigonal-pyramidal or a trigonal-planar geometry of the H-bound Si atom is present in **1H**, the theoretical studies suggest a flat energy hypersurface for the planarization of the three-coordinated Si atom.

**Table 2:** Comparison of selected experimental and calculated bonding parameters of **1H**, **1H_calc_** and **1H′_calc_**

	Si1–Si2 [Å]	Si1–C1 [Å]	Si2–C2 [Å]	∑_Si1_^*c*^ [°]	C1–Si1–Si2–C2 [°]	*φ*_NHC1_ ^*d*^ [°]	*φ*_NHC2_ ^*d*^ [°]
**1H**	2.281(3)	1.873(4)	1.873(4)	—	180.0(3)	3.3(2)	3.3(2)
**1H_calc_** ^*a*^	2.339	1.885	1.907	335.51	173.69	32.71	1.26
**1H_calc_** ^*b*^	2.308	1.861	1.884	342.58	173.63	21.95	3.41
**1H′_calc_** ^*b*^	2.289	1.841	1.886	359.61	179.32	6.68	3.24

^*a*^Calculated at the B3LYP/6-311G**/6-31G* level of theory.

^*b*^Calculated at the B97-D3/RI-JCOSX/def2-TZVP level of theory.

^*c*^∑_Si1_ is the sum of angles around the Si1 atom.

^*d*^ *φ*_NHC1_ and *φ*_NHC2_ denote the dihedral angles between the least-square plane of the atoms C1, Si1, Si2, C2 and the least square plane of the heterocyclic ring atoms of the NHC substituent bonded to Si1 and Si2, respectively.

**Fig. 6:**
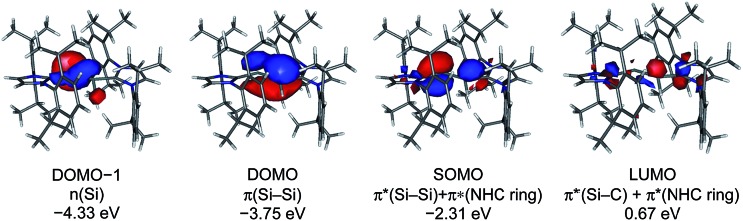
Quasi-restricted orbitals (QROs) of **1H_calc_** (B97-D3/RI-JCOSX/def2-TZVP) and their corresponding energy eigenvalues; isosurface value: 0.04 e bohr^–3^; DOMO = doubly occupied molecular orbital, SOMO = singly occupied molecular orbital, LUMO = lowest unoccupied molecular orbital.

The calculated quasi-restricted orbitals (QROs) of **1H_calc_** at the B3LYP/I level of theory and of **1H_calc_** and **1H′_calc_** at the B97-D3/II level of theory are almost identical (Fig. 6 and ESI, Fig. S17–S19†). The SOMO is the Si=Si π* orbital, confirming that reduction of **1H^+^** leads to a population of the empty Si=Si π* orbital of **1H^+^** with one electron (see ESI, Fig. S16†). The SOMO reveals significant contributions of π* NHC orbitals due to π-conjugation. The two lower lying doubly occupied molecular orbitals (DOMOs) are the Si=Si π and the *n*(Si) lone pair orbital, respectively.

Notably, CASSCF(3,3)/def2-TZVP calculations^26^ of **1H_calc_** revealed that the overall wave function is described by a major ground state configuration of [2-1-0] of the DOMO, SOMO and LUMO with 96% contribution, suggesting that static correlation can be neglected in the electronic description of **1H** (see ESI†).

**Fig. 7:**
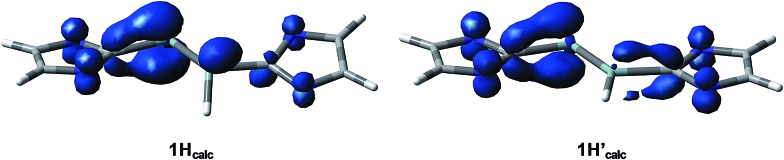
Spin densities of the calculated minimum structures **1H_calc_** (left) and **1H′_calc_** (right) at the B97-D3/RI-JCOSX/def2-TZVP level of theory. The N-bonded 2,6-diisopropylphenyl substituents are omitted for clarity.

The calculated spin densities of **1H_calc_** and **1H′_calc_** at the B97-D3/II level of theory are depicted in Fig. 7. Mulliken analyses^27^ of the spin densities reveal that the highest spin density is located at the dicoordinated Si2 atom (37% in **1H_calc_**, 29% in **1H′_calc_**), whereas the spin density at the Si1 atom is quite small (9% in **1H_calc_**, 6% in **1H′_calc_**), which is in full agreement with the observation of one large and one small *a*(^29^Si) hfcc in the experimental EPR spectrum of **1H** (*vide supra*) (see ESI, Table S9†).^28^ Remarkably, a significant amount of the spin density is delocalized into the C_NHC_ and N_NHC_ atoms of the Si1-bonded (17% in **1H_calc_**, 27% in **1H′_calc_**) and Si2-bonded (29% in **1H_calc_**, 30% in **1H′_calc_**) NHC substituents, which explains the EPR-spectroscopic detection of two *a*(^14^N) hfcc's. The calculated *g*_iso_ values of **1H_calc_** (2.00483) and **1H′_calc_** (2.00454) agree well with the experimentally obtained *g*_iso_ value (2.00562).

Further insight into the electronic structure of **1H** was provided by a natural bond orbital (NBO) analysis at the B3LYP/I level of theory (see ESI, Table S12†).25*k* The Si–Si bond is composed of a Si–Si σ bond and a Si=Si π bond with an occupancy of 1.95 and 0.82 electrons, respectively, which indicates indirectly a population of the Si=Si π* orbital with one electron leading thereby to a decrease of the formal Si–Si bond order from 2 in **1H^+^** to 1.5 in **1H** (*vide supra*). The Si2 atom in **1H_calc_** bears a lone pair of high s-character (72%) as similarly found for **1H^+^_calc_** (75%). Remarkably, both Si–C_NHC_ bonds in **1H_calc_** are composed of one doubly occupied Si–C_NHC_ σ NBO and one singly occupied Si=C_NHC_ π NBO, of which the latter is absent in **1H^+^_calc_**. These additional Si–C_NHC_ π contributions rationalize the shortening and strengthening of the Si–C_NHC_ bonds in **1H**, which is also reflected in the higher Si–C_NHC_ WBI indexes (**1H**: WBI(Si–C_NHC_) = 1.01 and 0.95; **1H^+^**: WBI(Si–C_NHC_) = 0.86 and 0.74).

Comparative analyses of the charge by natural population analyses (NPA) of **1H_calc_** and **1H^+^_calc_** at the B3LYP/I level of theory reveal that the positive partial charges at the Si atoms of **1H^+^_calc_** (*q*(Si1) = 0.27*e*, *q*(Si2) = 0.21*e*) are decreased by the reduction (**1H**: *q*(Si1) = 0.14*e*, *q*(Si2) = 0.03*e*) (see ESI, Table S13†). Furthermore, the one-electron reduction leads to a significant decrease of the overall charges of the NHC substituents (**1H^+^_calc_**: *q*(NHC1) = 0.36*e*, *q*(NHC2) = 0.30*e*; **1H**: *q*(NHC1) = 0.05*e*, *q*(NHC2) = –0.04*e*), whereas the hydridic character of the Si1-bonded H atom is retained (**1H^+^_calc_**: *q*(H) = –0.14*e*; **1H**: *q*(H) = –0.18*e*).

## 3. Conclusions

The isolation and full characterization of NHC-trapped Si_2_H (**1H**) can be considered as a major advance in low-valent silicon hydride chemistry, given the intermediacy of Si_2_H in the chemical vapor deposition of amorphous hydrogenated silicon that is widely used in solar cell and thin film transistors technology. Whereas Si_2_H features a *C*_2v_-symmetric H-bridged ground state structure and is a σ-type radical with a symmetric distribution of the spin density over the two silicon atoms, its NHC-trapped counterpart Si_2_(H)(Idipp)_2_ (**1H**) features a terminal Si–H bond and is a π-type radical, in which the unpaired electron occupies the Si=Si π* orbital (SOMO), leading to a formal Si–Si bond order of 1.5. Significant delocalization of the spin density into the NHC substituents occurs *via* π-conjugation of the Si=Si π* orbital with the π* orbitals of the coplanar arranged N-heterocyclic rings leading to a stabilization of the radical, in which the spin density is higher at the two-coordinated Si site. The mixed valent disilicon(0,I) hydride **1H** can be alternatively regarded as a H atom trapped in the closed shell compound Si_2_(Idipp)_2_. Implications of this view in hydrogen atom transfer chemistry^29^ are currently investigated.

## Supplementary Material

Supplementary information

Crystal structure data
