# Supplementary material for: The Si2H radical supported by two N-heterocyclic carbenes
Source: Chem Sci. 2016 May 9;7(8):4973–9. doi: 10.1039/c6sc01569g (PMC6018643; doi:10.1039/c6sc01569g)
Supplement: Supplementary file 1 [file SC-007-C6SC01569G-s001.pdf]

Electronic Supplementary Material (ESI) for Chemical Science.
This journal is © The Royal Society of Chemistry 2016

Electronic Supplementary Information

Table of contents

1. Experimental section – general part S2

2. Cyclic voltammetric studies of $[Si_2(H)(Idipp)_2][B(Ar^F)_4]$ (**1H**$[BAr^F)_4]$) S3

3. Synthesis of $Si_2(H)(Idipp)_2$ (**1H**) S5

3.1 Chemical one-electron oxidation of **1H** S5

4. UV-Vis-NIR spectroscopy of **1H** S7

5. Magnetic susceptibility measurement of **1H** S9

6. Single crystal X-ray diffraction analysis of **1H** S10

7. EPR spectroscopic analysis of **1H** S11

8. Quantum chemical calculations of **1H**$^+$ and **1H** S14

8.1 Comparison of selected experimental and calculated bonding parameters S15

8.2 Kohn-Sham orbitals of $\mathbf{1H}^+_{calc}$ and quasi-restricted orbitals of $\mathbf{1H}_{calc}$ and $\mathbf{1H'}_{calc}$ S17

8.3 Spin densities of $\mathbf{1H}_{calc}$ and $\mathbf{1H'}_{calc}$ S18

8.4 Comparison of experimental and calculated EPR parameters S19

8.5 Results of the natural bond order (NBO) calculations S19

8.6 Results of the TdDFT calculations S20

8.7 Results of the CASSCF calculations S24

8.8 Cartesian coordinates [Å] and SCF energies of the calculated structures S25

9. References S33

1. Experimental section – general part

All experiments were carried out under an atmosphere of argon using Schlenk or glove box techniques. Argon was commercially received with a purity of ≥99.999 % and passed through a gas purification system composed of two consecutive columns to remove traces of water and oxygen. The first column was filled with the BTS copper catalyst R3-11G from BASF and heated at ~80 °C and the second column with 4 Å molecular sieves. The glassware was dried in an oven at approximately 110 °C and baked under vacuum prior to use.

THF and *n*-hexane were refluxed several days over sodium wire/benzophenone and sodium wire/benzophenone/tetraglyme (0.5 vol%), respectively, purged several times with argon during reflux and distilled off under argon. Fluorobenzene and acetonitrile for the cyclic voltammetric measurements was stirred several days over CaH_2 and trap-to-trap condensed. Diethyl ether (Et_2O) used for the EPR samples was stirred several days over $LiAlH_4$ and trap-to-trap condensed. All solvents were degassed by three freeze-pump-thaw cycles and stored in the glove box.

The IR spectrum of **1H** (4000 – 400 cm^{-1}) was recorded on a Bruker Alpha FT-IR spectrometer in the glovebox with a diamond single-reflection Platinum-ATR module at room temperature. The following abbreviations were used for the intensities of the absorption bands: vs = very strong, s = strong, m = medium, w = weak, vw = very weak.

The C, H, N analysis of **1H** was carried out in triplicate on an Elementar Vario Micro elemental analyzer. The individual C, H, N values did not differ by more than ±0.3 %. The mean C, H, N values are given below for compound **1H**.

The thermal behaviour of compound **1H** was studied on a Büchi melting point B-545 apparatus. The samples were sealed in glass-capillary tubes under vacuum and heated once with a gradient of 5 K min^{-1} for a rough determination of the temperature of decomposition and twice with a gradient of 2 K min^{-1} (starting 20 K below the roughly determined temperature) for a precise determination of the temperature of decomposition. The molten samples were cooled to room temperature and studied by ^{1}H NMR spectroscopy verifying the decomposition of **1H**.

The compound $[Si_2(H)(Idipp)_2][B(Ar^F)_4]$ (**1H**$[B(Ar^F)_4]$, Idipp = $C[N(C_6H_3\text{-}2,6\text{-}i\text{Pr}_2)CH]_2$, Ar^F = $C_6H_3\text{-}3,5\text{-}(CF_3)_2$) was prepared as described recently.[S1] KC_8 was synthesized following the published procedure upon heating a 8:1 mixture of graphite and potassium to ca. 450 °C under vacuum.[S2] $[Fe(\eta^5\text{-}C_5Me_5)_2][B(Ar^F)_4]$ was synthesized as described in the literature and recrystallized from diethyl ether at −60 °C.[S3]

2. Cyclic voltammetric studies of $[Si_2(H)(Idipp)_2][B(Ar^F)_4]$ ($1H[B(Ar^F)_4]$)

The cyclic voltammetric studies were performed with an Autolab Eco electrochemical workstation composed of an Autolab PGSTAT 20 potentiostat/galvanostat. The results were analyzed with the Autolab software version 4.9. The experiments were carried out in a glove box under argon in a gas-tight specially designed full-glass three-electrode cell at ambient temperature. A glass-carbon disk electrode (Ø = 2 mm) was used as working electrode, a Pt wire of 1 mm diameter as counter electrode and a 4 mM $[Fe(C_5Me_5)_2]^{+1/0}$ / 0.1 M nBu$_4$NPF$_6$ solution as reference electrode, which was separated from the substrate/electrolyte solution with a Vycor frit (4 mm).[1] In all experiments fluorobenzene was used as solvent and tetra-*n*-butylammonium hexafluorophosphate (nBu$_4$NPF$_6$) as supporting electrolyte in a concentration of 0.1 M. The electrolyte was recrystallized twice from ethanol and dried for several hours at 150 °C before use. *iR*-drop compensation was applied for all experiments.

All potentials are reported relative to the reference electrode. For comparison reasons, the half-wave potential of the $[Fe(C_5H_5)_2]^{+1/0}$ versus the $[Fe(C_5Me_5)_2]^{+1/0}$ redox couple was determined under the same conditions by a separate cyclic voltammetric experiment and found to be $E_{1/2}$ = 520 mV.[2] For further comparison reasons, the half-wave potentials of the $[Fe(C_5H_5)_2]^{+1/0}$ and the $[Fe(C_5Me_5)_2]^{+1/0}$ redox couples in acetonitrile were determined versus the saturated calomel reference electrode (SCE), which was separated from the bulk solution by a salt bridge containing 0.1 M nBu$_4$NPF$_6$ in acetonitrile in order to modulate the SCE junction transport consequences,[S4] and found to be $E_{1/2}$ = 406 and −93 mV, respectively.

The cyclic voltammograms of **1** are depicted in Figures S1 and S2, and show that the compound undergoes a reversible reduction in fluorobenzene at a half-wave potential $E_{1/2}(1)$ of −1.626 V. The following criteria were applied to verify the reversibility of this process (Table S1):[S4]

a) the half-wave potential was found to be constant for several scan rates (v) ranging from 50 – 800 mV s^{-1};

b) the anodic and cathodic peak currents were almost identical (i_{pc} / i_{pa} ≈ 1) independent of the scan rate;

c) the difference between the cathodic and the anodic potentials ΔE_p ranged from 62 – 80 mV at scan rates of 50 – 800 mV s^{-1} and found to be slightly larger than the value for an

---

1 The $[Fe(C_5Me_5)_2]^{+1/0}$ redox couple was suggested to serve as a superior reference electrode compared to $[Fe(C_5H_5)_2]^{+1/0}$, see: a) I. Noviandri, K. N. Brown, D. S. Fleming, P. T. Gulyas, P. A. Lay, A. F. Masters, L. Phillips, *J. Phys. Chem. B* **1999**, *103*, 6713; b) J. R. Aranzaes, M.-C. Daniel, D. Astruc, *Can. J. Chem.* **2006**, *84*, 288.

2 $E_{1/2}$ corresponds to the thermodynamic standard redox potential of a redox pair, when the diffusion coefficients of the oxidized and reduced forms show little difference, which is often the case: J. Heinze, *Angew. Chem. Int. Ed. Engl.* **1984**, *23*, 831; *Angew. Chem.* **1984**, *96*, 823.

ideal Nernstian process (58 mV). The deviation of ΔE_p from that of an ideal Nernstian process can be rationalized by an incomplete iR drop compensation;

d) a plot of the cathodic peak current i_{pc} against the square root of the scan rate confirmed an almost linear relationship (Figure S3).

One irreversible oxidation was also observed at $E_{1/2}(2)$ = 0.665 V at a scan rate of 100 mV s^{-1}, for which the cathodic peak could not be sufficiently resolved. The ratio of the cathodic and anodic peak currents was significantly smaller than 1 and the difference between the cathodic and the anodic potentials of 86 mV was significantly larger than 58 mV, thus indicating a rapid follow-up chemical reaction of a putative $[Si_2(H)(Idipp)_2]^{2+}$ dication.

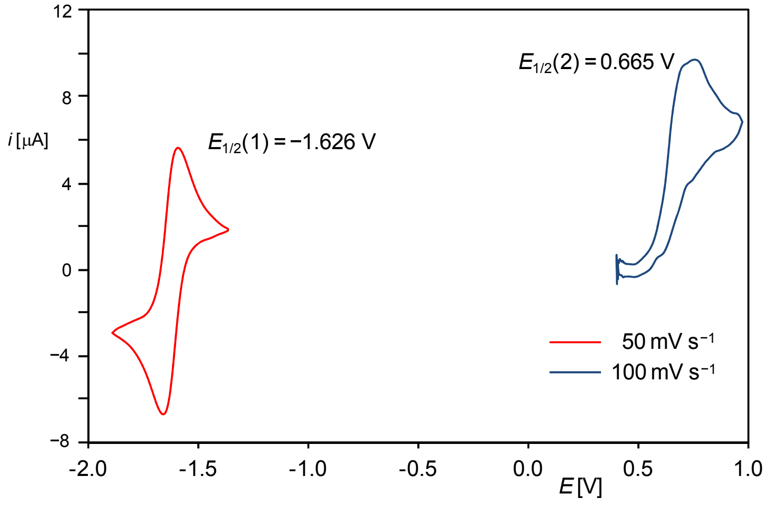

Figure S1. Single-scan cyclic voltammograms of **1H**[B(Ar^F)$_4$] in fluorobenzene from (−2.000) – 1.000 V at ambient temperature at scan rates of 50 and 100 mVs^{-1}; reference electrode: 4 mM $[Fe(C_5Me_5)_2]^{+1/0}$ / 0.1 M nBu$_4$NPF$_6$ in fluorobenzene; electrolyte: 0.1 M nBu$_4$NPF$_6$.

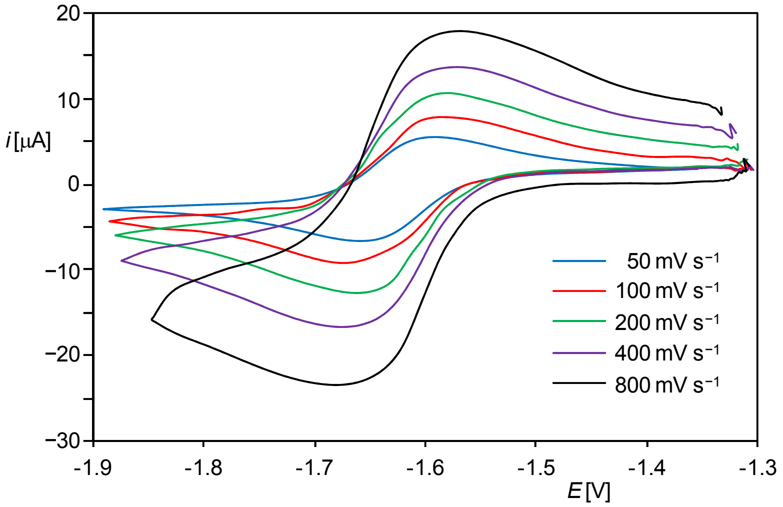

Figure S2. Single-scan cyclic voltammograms of **1H**[B(Ar^F)$_4$] in fluorobenzene from (−1.900) – (−1.300) V at ambient temperature at different scan rates (50 – 800 mV s^{-1}); reference electrode: 4 mM $[Fe(C_5Me_5)_2]^{+1/0}$ / 0.1 M nBu$_4$NPF$_6$ in fluorobenzene; electrolyte: 0.1 M nBu$_4$NPF$_6$.

Table S1: Results of the cyclic voltammetric analysis of **1H**[B(Ar^F)$_4$].[a]

v [mV s^{-1}]	ΔE_p [mV]	i_{pc} / i_{pa}	$E_{1/2}$ [V]
50	62	1.00	−1.627
100	72	1.03	−1.628
200	76	1.00	−1.624
400	80	1.01	−1.624
600	66	1.03	−1.625
800	70	1.04	−1.627
100	86	0.20	0.665

[a]: v = scan rate; $\Delta E_p = E_{pa} - E_{pc}$, where E_{pa} is the anodic peak potential and E_{pc} the cathodic peak potential; i_{pc} / i_{pa} = ratio of cathodic and anodic peak current; $E_{1/2} = (E_{pa} + E_{pc})$ / 2 = half-wave potential. Potentials are given versus the [Fe(C$_5$Me$_5$)$_2$]$^{+1/0}$ redox pair.

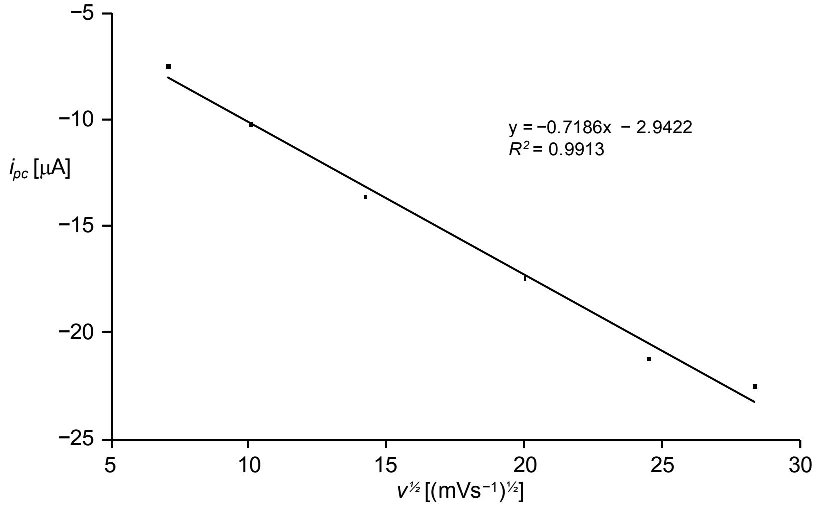

Figure S3. Plot of the cathodic peak current (i_{pc}) against the square root of the scan rate ($v^{½}$) for the reversible reduction of **1H**[B(Ar^F)$_4$] at $E_{1/2}$ = −1.626 V.

3. Synthesis of Si$_2$(H)(Idipp)$_2$ (1H)

To a mixture of compound **1H**[B(Ar^F)]$_4$ (250 mg, 0.147 mmol) and KC$_8$ (22 mg, 0.162 mmol, 1.1 eq.) were added 4 mL of THF by vacuum transfer at −196 °C. The mixture was warmed to −40 °C and a brownish suspension was obtained. Stirring for 10 minutes afforded a green solution with a black precipitate, which was further stirred at −40 °C for 30 minutes. The suspension was warmed to room temperature and stirred for 1 h. Subsequent evaporation of the solvent under vacuum afforded a dark green-black residue, which was dried for 30 minutes under vacuum. The solid was extracted with 3 × 5 mL and 1 × 2 mL of *n*-hexane, of which the last extract had only a slightly greenish color. The combined dark green-blue extracts were concentrated under vacuum to ca. 3 mL (incipient precipitation of a dark green solid) and stored at −60 °C for 48 h. The dark green solid was separated from the mother liquor with a filter cannula at −60 °C and dried for 1 h at room temperature. Yield: 67 mg (0.08 mmol, 55 %). Elemental analysis calcd (%) for C$_{54}$H$_{73}$N$_4$Si$_2$ (834.36): C 77.73, H

8.82, N 6.71; found: C 76.00 H 8.54 N 6.31 %. Melting point: 147 °C (decomposition upon melting to a dark red mass).[3]

IR (solid, RT, Figures S4 and S5): ν [cm^{-1}] =409 (w), 460 (w), 495 (w), 521 (vw), 548 (w), 584 (vw), 638 (w), 670 (w), 694 (s), 714 (vw), 729 (vw), 750 (m), 757 (m), 794 (s), 839 (vw), 886 (vw), 930 (w), 958 (w), 1040 (s), 1074 (vs), 1083 (vs), 1113 (m), 1147 (vw), 1165 (vw), 1178 (w), 1200 (m), 1229 (vs), 1244 (vs), 1272 (m), 1292 (m), 1302 (m), 1315 (w), 1330 (w), 1360 (m), 1385 (m), 1457 (m), 1466 (m), 1496 (vw), 1553 (vw), 1590 (vw), 1609 (vw), 2089 (w) [ν(Si–H)], 2864 (m), 2924 (m), 2958 (m), 3030 (vw), 3067 (vw), 3130 (vw).

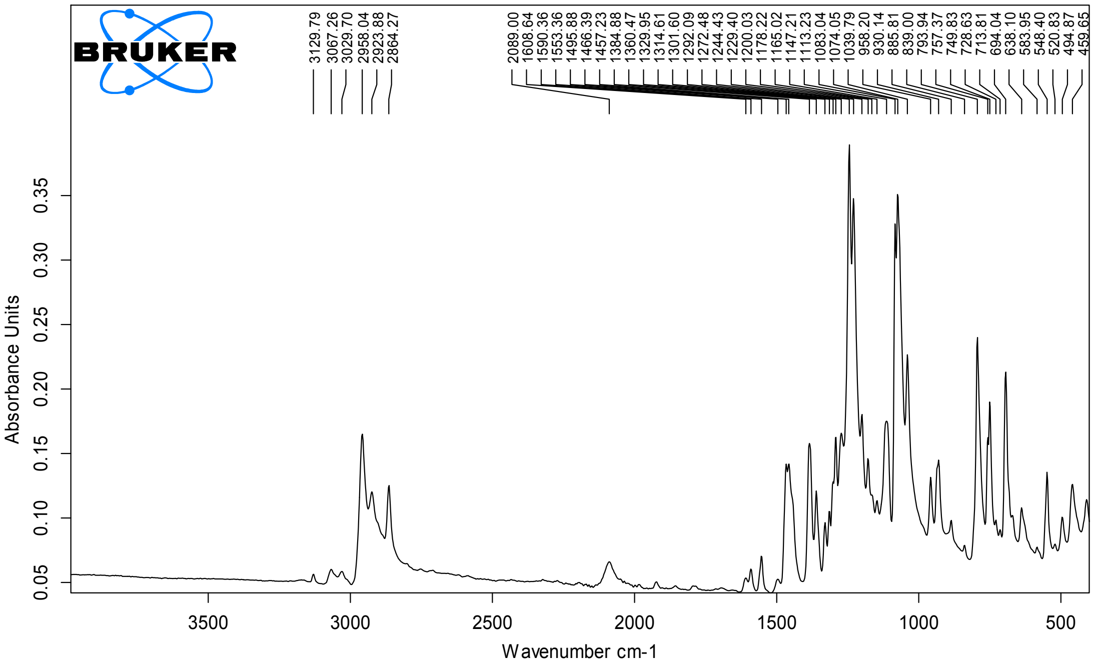

Figure S4. ATR FT-IR spectrum of a solid state sample of **1H**.

3 A ^{1}H NMR spectrum of the red mass obtained after cooling of the molten sample to room temperature in C_6D_6 showed the presence of Idipp and a small amount (ca. 5 %) of $Si_2(Idipp)_2$.

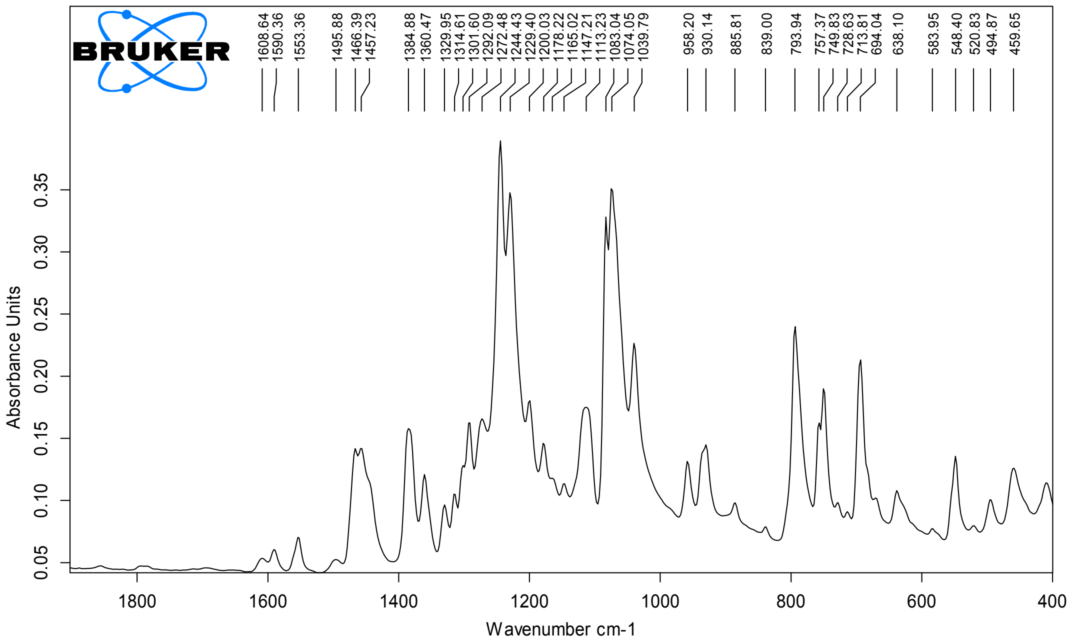

Figure S5. Excerpt of the ATR FT-IR spectrum of a solid state sample of **1H** from 400 – 1900 cm^{-1}.

3.1 Chemical one-electron oxidation of 1H

A J. Young NMR tube was charged with 10 mg (12 μmol) of **1H** and 14 mg (12 μmol, 1.0 eq.) of $[Fe(\eta^5\text{-}C_5Me_5)_2][B(Ar^F)_4]$. Upon addition of 0.5 mL of THF-d_8 a dark green solution was obtained, which rapidly turned dark red. The recorded ^1H NMR spectrum showed the signals of **1H**$[B(Ar^F)_4]$ and $[Fe(\eta^5\text{-}C_5Me_5)_2]$, indicating a selective one-electron oxidation of **1H** to **1H**$[B(Ar^F)_4]$.

4. UV-Vis-NIR spectroscopy of 1H

The UV-Vis-NIR spectra of **1H** were recorded on a Thermo Scientific Evolution 300 spectrometer in a special designed quartz cuvette under inert conditions. The measurements were performed using three different path lengths (d = 1 mm, 5 mm and 10 mm) and different concentrations (c = 360 μmol L^{-1} and 250 μmol L^{-1}) in n-hexane at ambient temperature. The UV-Vis-NIR spectra are depicted in Figure S6 and the absorption maxima and corresponding molar extinction coefficients ε_λ are summarized in Table S2. The absorption bands were determined by means of band deconvolution assuming a Gaussian line profile. The corresponding deconvoluted absorption bands are displayed in Figure S7 and the parameters used for the band deconvolution are summarized in Table S3.[4]

[4] The measured spectrum was approximated as a convolution of different Gaussian functions, which represent the single absorption bands. The deconvolution operation was performed using Microsoft Excel 2007 following standard methods (see for example: E. J. Billo, *Excel for chemists - A comprehensive guide,* 3rd ed., Wiley-VCH, Weinheim, **2011**, pp. 474–476).

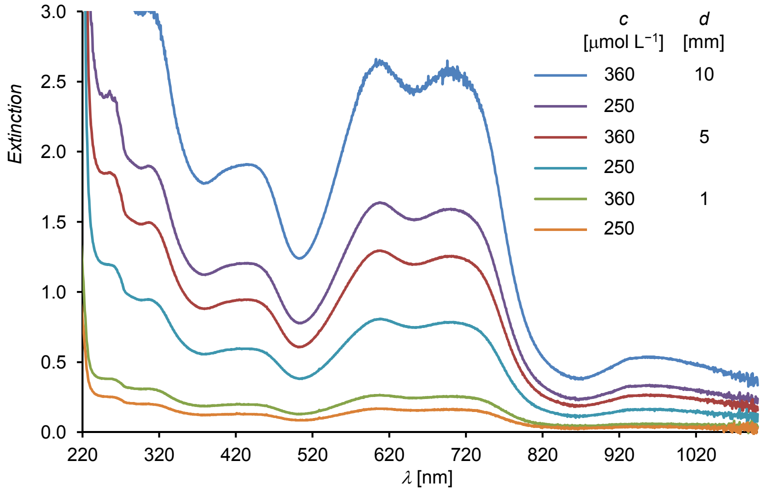

Figure S6. Experimental UV-Vis-NIR spectra of **1H** in *n*-hexane from 220 – 1100 nm at different concentrations *c* of **1H** and path lenghts *d* of the cuvette.

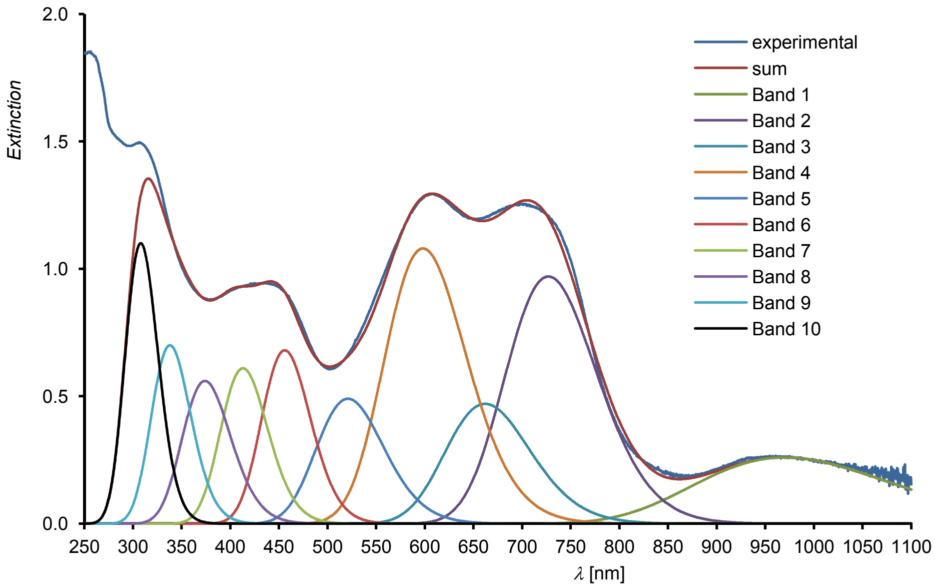

Figure S7. Deconvoluted and experimental UV-Vis-NIR spectra of **1H** in *n*-hexane at *c* = 360 μmol L^{-1} and *d* = 5 mm.

Table S2: Absorption maxima of the UV-Vis-NIR spectra of **1H** depicted in Figure S6 and their corresponding molar extinction coefficients.

λ [nm]	254	305	436	608	704	958
$\varepsilon_\lambda \cdot 10^{-3}$ [L mol^{-1} cm^{-1}]	9.97	8.14	5.17	7.11	6.86	1.44

Table S3: Parameters used for the band deconvolution of the UV-Vis-NIR spectrum depicted in Figure S7.[a]

	Band 1	Band 2	Band 3	Band 4	Band 5	Band 6	Band 7	Band 8	Band 9	Band 10
λ	970	727	662	598	521	456	413	374	338	308
E	0.26	0.97	0.47	1.08	0.49	0.68	0.61	0.56	0.70	1.10
σ	1500	1250	1400	1650	1800	1700	2000	2500	2500	2500

[a]: λ_{max} = wavelength [nm]; E = extinction maximum; σ = linewidth [cm^{-1}].

5. Magnetic susceptibility measurement of 1H

The magnetic susceptibility measurement of **1H** was performed on a PPMS VLM-409p vibrating-sample magnetometer in a temperature range of 1.9 – 300 K and the obtained susceptibilities were diamagnetically corrected.[S5]

A plot of the reciprocal molar magnetic susceptibility against the absolute temperature (Figure S8) shows a linear correlation as expected for a paramagnetic compound following Curie's law.[S6] Calculation of the effective magnetic moment from the slope of the line obtained after linear regression (R^2 = 0.9903) yielded μ_{eff} = 1.68μ_{B} (μ_B = Bohr magneton = 9.27400968(20) · 10^{-24} J T^{-1}). The value is slightly lower than the expected value derived from the spin-only formula for one unpaired electron (μ_{eff} = 1.73μ_B).[S6] The difference might arise from a slight decomposition of the extremely air-sensitive compound **1H** during the measurement. A plot of the effective magnetic moment against the absolute temperature (Figure S9) revealed only a small temperature dependence of the effective magnetic moment of **1H**. The obtained data clearly suggest the presence of one unpaired electron in compound **1H**.

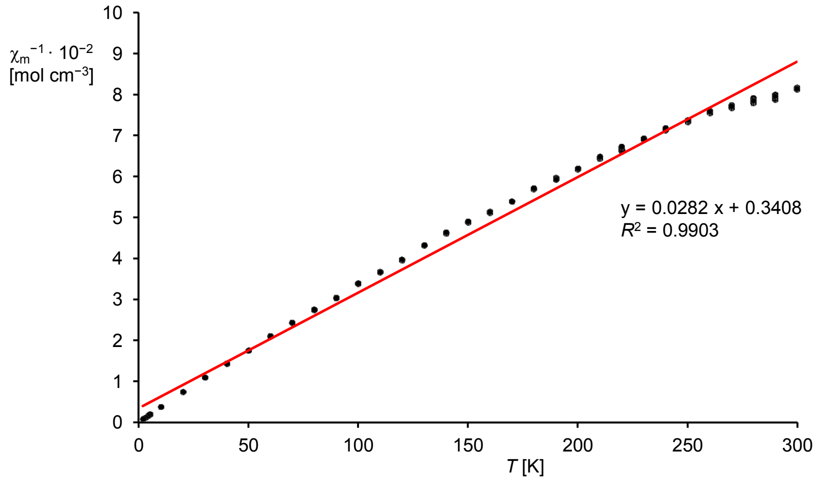

Figure S8. Plot of the reciprocal molar magnetic susceptibility χ_{m}^{-1} against the absolute temperature T (dotted black line) and the corresponding line (red) and line equation obtained by linear regression.

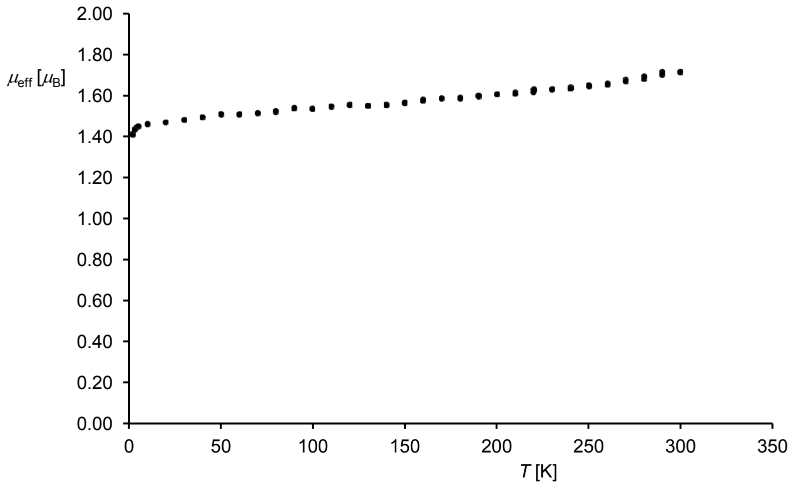

Figure S9. Plot of the effective magnetic moment μ_{eff} against the absolute temperature T; μ_B = Bohr magneton = 9.27400968(20) · 10^{-24} J T^{-1}.

6. Single crystal X-Ray diffraction analysis of 1H

Clear dark blue blocks of **1H** suitable for single crystal X-ray diffraction were obtained upon crystallization from a concentrated *n*-hexane solution at −60 °C. The crystals were protected with Fomblin® Y during mounting on the goniometer.

The data collection was performed on a STOE IPDS-2T diffractometer using graphite monochromated Mo-K_α radiation (λ = 0.7107 Å). The diffractometer was equipped with a low-temperature device (Oxford Cryostream 700er series, 123(2) K). Intensities were measured by fine-slicing ω and φ-scans and corrected for background, polarization and Lorentz effects. An absorption correction by integration was applied for all data sets.[S7] The structures were solved by direct methods and refined anisotropically by the least-squares procedure implemented in the SHELX program system.[S8] Hydrogen atoms except the silicon-bonded hydrogen atom were included using the riding model on the bound carbon atoms. The silicon-bonded hydrogen atom was found on the difference Fourier map and anisotropically refined with an occupancy of 1/2, which results from the inversion centre of the space group $P2_1/c$. Due to the low electronic density, the position of the silicon-bonded hydrogen atom cannot be accurately determined by X-ray diffraction analysis. This was verified by changing the position of the hydrogen atom, including a bridged position between the silicon atoms, which resulted in no variation of the final R indices. Selected crystallographic refinement data are listed in Table S4.

CCDC-1471165 contains the supplementary crystallographic data for this paper, which can be obtained free of charge from the Cambridge Crystallographic Data Centre via www.ccdc.cam.ac.uk/data_request/cif.

Table S4: Crystal data and structure refinement of **1H**.

Empirical formula	$C_{54}H_{73}N_4Si_2$
Moiety formula	$C_{54}H_{73}N_4Si_2$
Formula weight	834.36 g mol^{-1}
Temperature	123(2) K
Wavelength	0.71073 Å
Crystal system, space group	monoclinic, $P2_1/c$
Unit cell dimensions	a = 12.9588(11) Å, α = 90°
	b = 13.5352(9) Å, β = 126.432(5)°
	c = 18.2226(15) Å, γ = 90°
Volume	2571.6(3) Å^3
Z	2
ρ_{calc}	1.078 mg m^{-3}
μ	0.106 mm^{-1}
F(000)	906.0
Crystal size	0.24 × 0.18 × 0.12 mm
2θ-range for data collection	5.42 – 56°
Limiting indices	$-17 \le h \le 17$, $-17 \le k \le 17$, $-24 \le l \le 24$
Reflections collected	20621
Independent reflections	6197 (R_{int} = 0.1649, R_σ = 0.2106)
Completeness to θ	99.9 %
Absorption correction	integration
Min. and max. transmission	0.6417; 0.9185
Refinement method	Full-matrix least squares on F^2
Data / restraints / parameters	6197 / 0 / 279
Goodness-of-fit on F^2	0.754
Final R indices [$I \ge 2\sigma(I)$]	R_1 = 0.0754, wR_2 = 0.1719
Final R indices (all data)	R_1 = 0.1884, wR_2 = 0.2041
Largest diff. peak / hole	0.46 / −0.27 e Å^{-3}

7. EPR spectroscopic analysis of 1H

The samples used for the EPR experiments were prepared by dissolving a small amount of solid **1H** (approximately 1 mg) in 4 mL of *n*-hexane or diethyl ether in the glove box to afford a green colored solution ($c \approx 300$ μmol L^{-1}). The solution was transferred to a Wilmad® Suprasil EPR tube (Ø = 3.8 mm) with a syringe and the tube was sealed off under vacuum with an oxygen/hydrogen burner.

The continuous-wave (cw) EPR experiments were performed in *n*-hexane in the temperature range of 183 – 336 K at X-band microwave (MW) frequencies on a Bruker EMXmicro EPR spectrometer with the EMXmicro standard resonator. The sample temperature was adjusted using a liquid nitrogen evaporator and the ER 4131VT temperature control system.

The EPR experiment in diethyl ether was performed on a Bruker ELEXSYS E580 EPR spectrometer. A Super High-Q resonator and an Oxford ESR900 helium gas-flow cryostat was employed for the cw measurement at X-band MW frequency.

For each measurement it was validated that neither a saturation of the EPR signal occurred nor that the resolution could be further improved by varying the microwave power and the modulation amplitude, respectively.

The measurement parameters are given in Table S5. The spectra were simulated using the garlic routine of the EasySpin program package.[S9] The simulation parameters for all spectra are given in Tables S6 and S7.

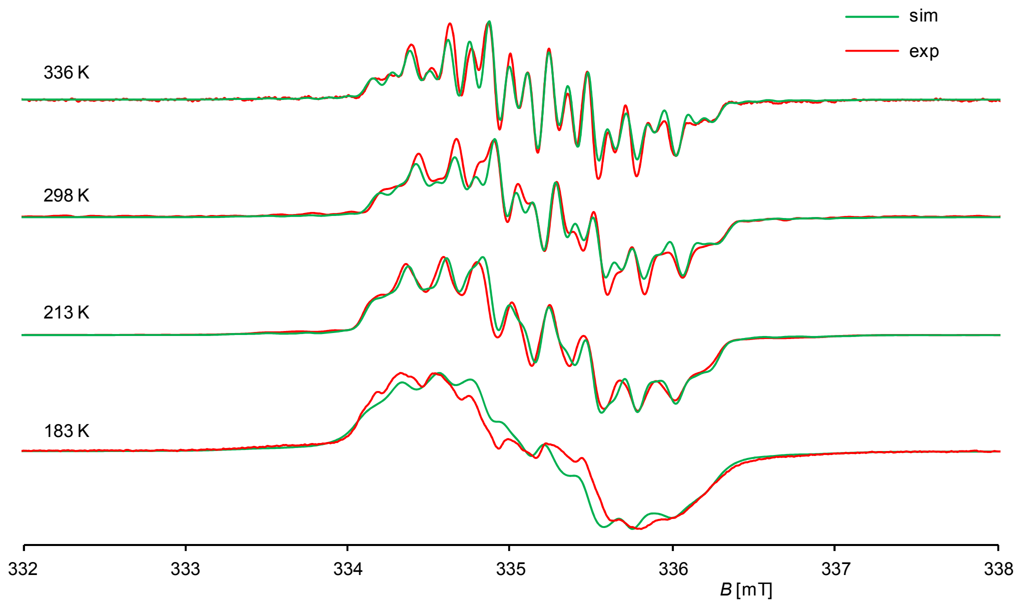

Figure S10. Experimental (red curves) and simulated (green curves) X-band EPR spectra of **1H** in *n*-hexane from 183 – 336 K; the ordinate (d*A*/d*B*) is omitted for clarity.

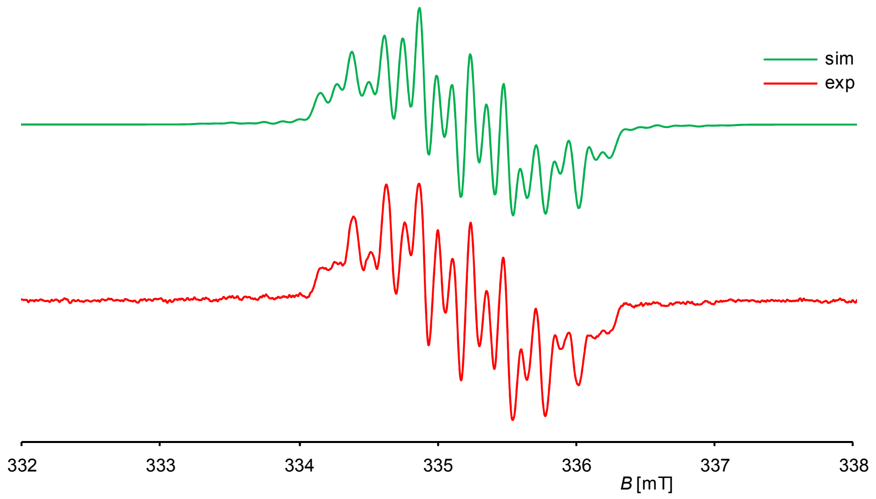

Figure S11. Experimental (bottom, red curve) and simulated (top, green curve) X-band EPR spectra of **1H** in *n*-hexane at 336 K; the ordinate (d*A*/d*B*) is omitted for clarity.

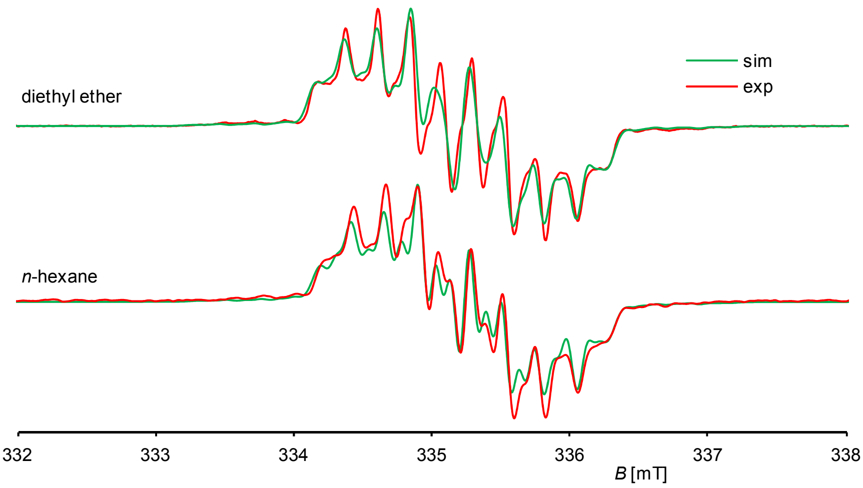

Figure S12. Experimental (red curves) and simulated (green curves) X-band EPR spectra of **1H** in diethyl ether (top) and *n*-hexane (bottom) at 298 K; the ordinate (d*A*/d*B*) is omitted for clarity.

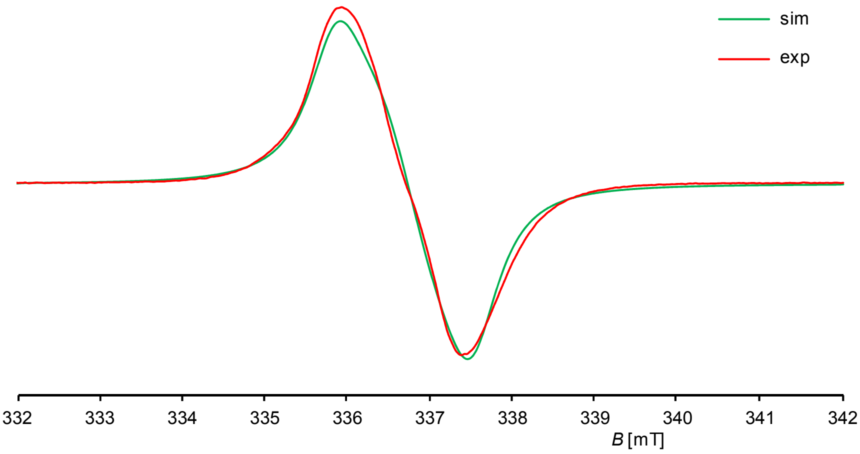

Figure S13. Experimental (red curve) and simulated (green curve) X-band EPR spectra of **1H** in frozen *n*-hexane solution at 153 K; the ordinate (d*A*/d*B*) is omitted for clarity.

Table S5: Parameters used for the EPR measurements of **1H**.[a]

solvent	*T* [K]	*ν* [GHz]	*MA* [G]	*P* [mW]	*RG* [d*B*]	*CT* [ms]	*TC* [ms]	*CF* [mT]	*SW* [mT]	*NP*
n-hexane	153	9.4523	1.0	0.578	30	32.10	20.48	337.0	10.0	1902
n-hexane	183	9.4542	0.2	0.578	30	6.41	20.48	337.0	10.0	9509
n-hexane	213	9.4491	1.0	0.057	30	32.10	20.48	337.0	10.0	1902
n-hexane	298	9.4591	1.0	0.057	30	32.10	20.48	337.0	10.0	1902
n-hexane	336	9.4491	0.2	0.057	30	6.44	20.48	337.0	10.0	9509
diethyl ether	298	9.4094	0.5	1.262	40	21.00	20.48	335.5	10.0	1024

[a]: *T* = temperature, *ν* = microwave frequency, *MA* = modulation amplitude, *P* = microwave power, *RG* = receiver gain, *CT* = conversion time, *TC* = time constant, *CF* = center field, *SW* = sweep width, *NP* = number of points on the field axis.

Table S6: Isotropic *g* values, hyperfine coupling constants (*a*) and lineshape parameters used to simulate the spectrum of **1H** in liquid *n*-hexane and diethyl ether solution.[a]

solvent	T [K]	g	$a(^{29}Si1)$ [mT]	$a(^{29}Si2)$ [mT]	$a(^{14}N1)$ [mT]	$a(^{14}N2)$ [mT]	$a(^{1}H)$ [mT]	$lwpp_G$ [mT]	$lwpp_L$ [mT]
n-hexane	183	2.00585	1.725	0.431	0.246	0.099	0.630	0.11	0.08
n-hexane	213	2.00567	1.725	0.431	0.244	0.097	0.622	0.11	0.02
n-hexane	298	2.00538	1.725	0.431	0.246	0.099	0.607	0.10	0.02
n-hexane	336	2.00562	1.725	0.431	0.246	0.100	0.605	0.08	0.02
diethyl ether	298	2.00553	1.775	0.444	0.246	0.098	0.657	0.11	0.01

[a]: T = temperature; $lwpp_G$ = linewidth peak-to-peak for the Gaussian contribution; $lwpp_L$ = linewidth peak-to-peak for the Lorentzian contribution.

Table S7: Anisotropic *g* values and lineshape parameters used to simulate the spectrum of **1H** in frozen *n*-hexane solution.[a]

solvent	T [K]	g_{11}	g_{22}	g_{33}	g_{iso}	$lwpp_G$ [mT]	$lwpp_L$ [mT]	HS_{11} [mT]	HS_{22} [mT]	HS_{33} [mT]
n-hexane	153	2.00110	2.00550	2.01070	2.00577	0.13	0.46	0.04	0.53	0.04

[a]: T = temperature; $lwpp_G$ = linewidth peak-to-peak for the Gaussian contribution; $lwpp_L$ = linewidth peak-to-peak for the Lorentzian contribution, HS = hyperfine strain parameter. The isotropic *g* value (g_{iso}) was calculated according to $g_{iso} = (g_{11} + g_{22} + g_{33})/3$.

8. Quantum-chemical calculations of $1H^+$ and 1H

The DFT calculations of the compounds $\mathbf{1H^+}$ and **1H** were carried out using the B3LYP functionals[S10] in combination with the 6-311G** basis set[S11] for the Si, N, Si-bonded H and heterocyclic ring C atoms and the 6-31G* basis set for the peripherical C and H atoms or the B97-D3 functionals[S12] in combination with the RI-JCOSX approximations[S13] and the def2-TZVP basis sets[S14] for all atoms. The structure optimizations were performed without symmetry restraints using the ORCA 3.0.0 program package with its internal standard convergence criteria.[S15] The optimized geometries were verified as minima on the potential energy surface by two-sided numerical differentiation of the analytical gradients to obtain harmonic frequencies, which were also used to calculate the zero point vibrational energies (ZPVE). The calculations of the EPR parameters were carried out at the B97-D3/RI-JCOSX/def2-TZVP level of theory. The spin densities were obtained by a Mulliken spin density analysis at the B97-D3/RI-JCOSX/def2-TZVP level of theory.[S16] The natural bond orbital (NBO) analyses were performed using the NBO 3.1 program at the B3LYP/6-311G**/6-31G* level of theory.[S17]

8.1 Comparison of selected experimental and calculated bonding parameters

Calculations at the B3LYP/6-311G**/6-31G* level of theory revealed one optimized minimum structure for **1H$_{calc}$** with a pyramidalized Si1 atom (Figure S14, Table S8). For comparison reasons, the bonding parameters of the optimized minimum structure of **1H$^{+}_{calc}$** were also calculated at the B3LYP/6-311G**/6-31G* level of theory (Figure S14, Table S8). Calculations at the B97-D3/RI-JCOSX/def2-TZVP level of theory led to two structurally different minimum structures (Figure S15, Table S8). The first minimum structure (**1H$_{calc}$**) features a pyramidalized Si1 atom and shows similar bonding parameters as the minimum structure calculated at the B3LYP/6-311G**/6-31G* level of theory (Table S8). The second minimum structure (**1H'$_{calc}$**), which is 5.5 kJ mol^{-1} higher in energy compared to **1H$_{calc}$**, consists of a trigonal-planar Si1 atom and a more coplanar orientation of the Si1-bonded NHC substituent, whereas the other bonding parameters are similar to those of **1H$_{calc}$** (Table S8).

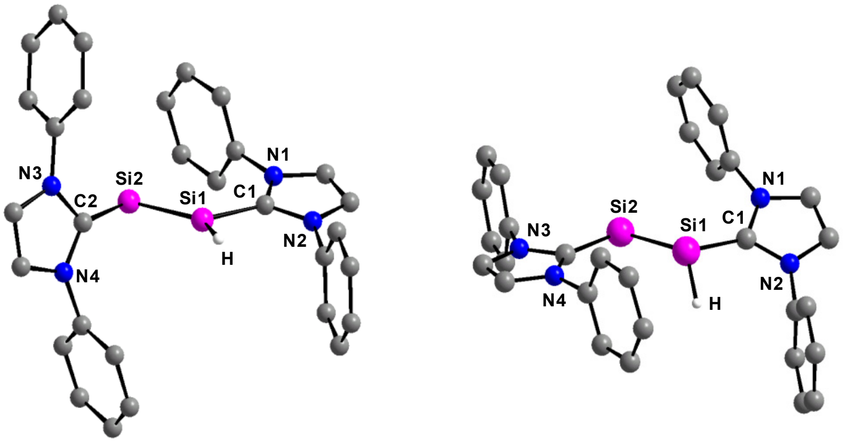

1H$^{+}_{calc}$ **1H$_{calc}$**

Figure S14. Calculated (**1H$^{+}_{calc}$** and **1H$_{calc}$**, B3LYP/6-311G**/6-31G*) structures of $[Si_2(H)(Idipp)_2]^+$ and $Si_2(H)(Idipp)_2$. The H atoms, except the H atoms bonded to Si1, and the *i*Pr substituents are omitted for clarity.

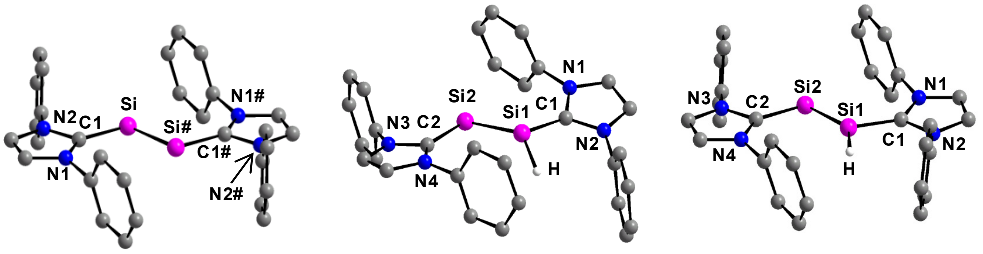

1H **1H$_{calc}$** (0.0 kJ mol^{-1}) **1H'$_{calc}$** (5.5 kJ mol^{-1})

Figure S15. Experimental (**1H**) and calculated (**1H$_{calc}$** and **1H'$_{calc}$**, B97-D3/RI-JCOSX/def2-TZVP) structures of $Si_2(H)(Idipp)_2$. The relative energies of **1H$_{calc}$** and **1H'$_{calc}$** are given in brackets. The H atoms, except the H atom bonded to Si1 in **1H$_{calc}$** and **1H'$_{calc}$**, and the *i*Pr substituents are omitted for clarity.

Table S8: Comparison of selected experimental and calculated bonding parameters of **1H**$^{+}$ in **1H**[B(Ar^{F})$_4$], **1H**$^{+}_{calc}$, **1H**, **1H**$_{calc}$ and **1H'**$_{calc}$.

	Si1–Si2 [Å]	Si1–C1 [Å]	Si2–C2 [Å]	Si1–H [Å]	C1–N1 [Å]	C1–N2 [Å]	C2–N3 [Å]	C2–N4 [Å]
**1H**$^{+}$[a]	2.1873(8)	1.882(2)	1.940(2)	1.32(2)	1.356(2)	1.358(2)	1.356(2)	1.358(2)
**1H**$^{+}_{calc}$[b]	2.209	1.896	1.972	1.481	1.369	1.366	1.366	1.366
**1H**	2.281(3)	1.873(4)	1.873(4)	–	1.381(4)	1.402(4)	1.381(4)	1.402(4)
**1H**$_{calc}$[b]	2.339	1.885	1.907	1.496	1.392	1.389	1.400	1.397
**1H**$_{calc}$[c]	2.308	1.861	1.884	1.495	1.393	1.388	1.397	1.393
**1H'**$_{calc}$[c]	2.289	1.841	1.886	1.489	1.399	1.392	1.400	1.393
	C1-Si1-Si2 [°]	C2-Si2-Si1 [°]	C1-Si1-H [°]	Si2-Si1-H [°]	Σ_{Si1}[d] [°]	C1-Si1-Si2-C2 [°]	φ_{NHC1}[f] [°]	φ_{NHC2}[f] [°]
**1H**$^{+}$[a]	116.73(7)	95.34(6)	106(1)	138(1)	360(1)[e]	177.61(9)	8.60(6)	71.06(6)
**1H**$^{+}_{calc}$[b]	120.12	97.94	103.60	136.11	359.83	−177.26	12.07	73.46
**1H**	109.5(1)	109.5(1)	–	–	–	180.0(3)	3.3(2)	3.3(2)
**1H**$_{calc}$[b]	112.09	104.20	98.90	124.52	335.51	173.69	32.71	1.26
**1H**$_{calc}$[c]	111.88	102.40	99.87	130.83	342.58	173.63	21.95	3.41
**1H'**$_{calc}$[c]	116.55	102.87	101.90	141.16	359.61	179.32	6.68	3.24

[a]: Experimental structural parameters obtained from ref. [S1]. [b]: Calculated at the B3LYP/6-311G**/6-31G* level of theory. [c]: Calculated at the B97-D3/RI-JCOSX/def2-TZVP level of theory. [d]: Σ_{Si1} is the sum of angles around the Si1 atom. [e]: The uncertainty (u) of the sum of angles is given in parenthesis and was calculated from the individual uncertainties (u_i) by error propagation using the formula $u = (\Sigma(u_i)^2)^{1/2}$. [f]: φ_{NHC1} and φ_{NHC2} denote the dihedral angles between the least-square plane of the atoms C1, Si1, Si2, C2 and the least square plane of the heterocyclic ring atoms of the NHC substituent bonded to Si1 and Si2, respectively.

8.2 Kohn-Sham orbitals of $1H^{+}_{calc}$ and quasi-restricted orbitals of $1H_{calc}$ and $1H'_{calc}$

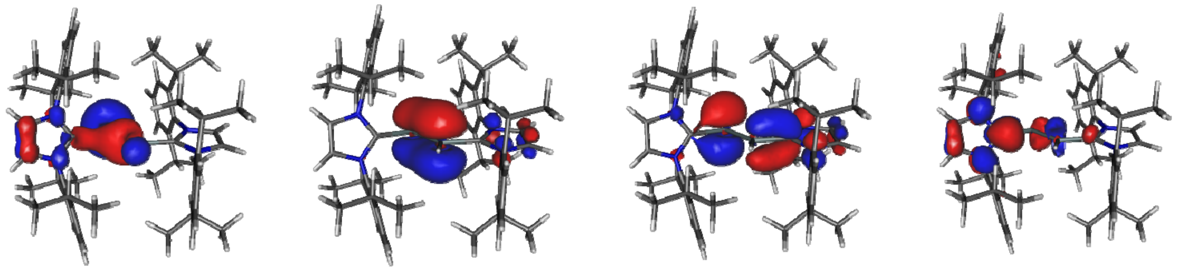

HOMO−1	HOMO	LUMO	LUMO+1
n(Si)	π(Si–Si)	π*(Si–Si) + π*(NHC ring)	π*(NHC ring)
−7.84 eV	−7.05 eV	−4.07 eV	−2.98 eV

Figure S16. Kohn-Sham orbitals of $\mathbf{1H^{+}_{calc}}$ (B3LYP/6-311G**/6-31G*) and their corresponding energy eigenvalues; isosurface value: 0.04 e bohr^{-3}; HOMO = highest occupied molecular orbital, LUMO = lowest unoccupied molecular orbital.

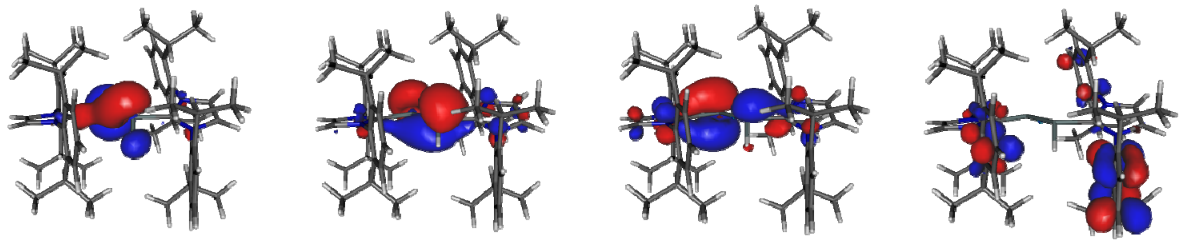

DOMO−1	DOMO	SOMO	LUMO
n(Si)	π(Si–Si)	π*(Si–Si)+π*(NHC ring)	π*(NHC aryl) and π*(NHC ring
−4.45 eV	−3.39 eV	−1.24 eV	−0.14 eV

Figure S17. Quasi-restricted orbitals (QROs) of $\mathbf{1H_{calc}}$ (B3LYP/6-311G**/6-31G*) and their corresponding energy eigenvalues; isosurface value: 0.04 e bohr^{-3}; DOMO = doubly occupied molecular orbital, SOMO = singly occupied molecular orbital, LUMO = lowest unoccupied molecular orbital.

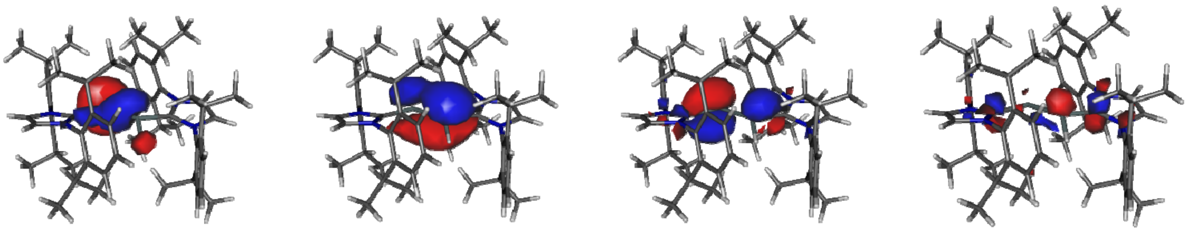

DOMO−1	DOMO	SOMO	LUMO
n(Si)	π(Si–Si)	π*(Si–Si)+π*(NHC ring)	π*(Si–C) + π*(NHC ring)
−4.33 eV	−3.75 eV	−2.31 eV	0.67 eV

Figure S18. Quasi-restricted orbitals (QROs) of $\mathbf{1H_{calc}}$ (B97-D3/RI-JCOSX/def2-TZVP) and their corresponding energy eigenvalues; isosurface value: 0.04 e bohr^{-3}; DOMO = doubly occupied molecular orbital, SOMO = singly occupied molecular orbital, LUMO = lowest unoccupied molecular orbital.

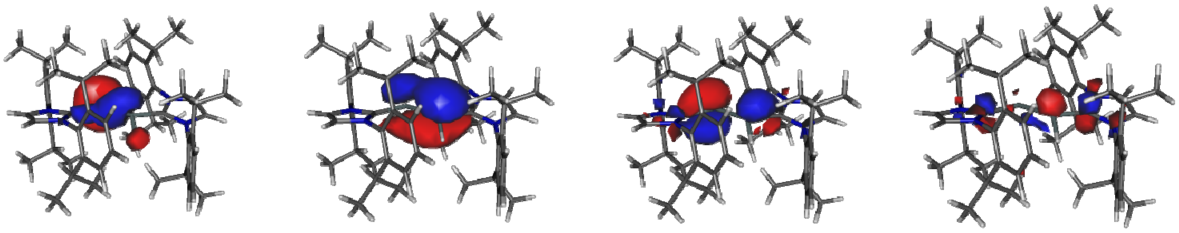

Figure S19. Quasi-restricted orbitals (QROs) of **1H’$_{calc}$** (B97-D3/RI-JCOSX/def2-TZVP) and their corresponding energy eigenvalues; isosurface value: 0.04 e bohr^{-3}; DOMO = doubly occupied molecular orbital, SOMO = singly occupied molecular orbital, LUMO = lowest unoccupied molecular orbital.

8.3 Spin densities of 1H$_{calc}$ and 1H’$_{calc}$

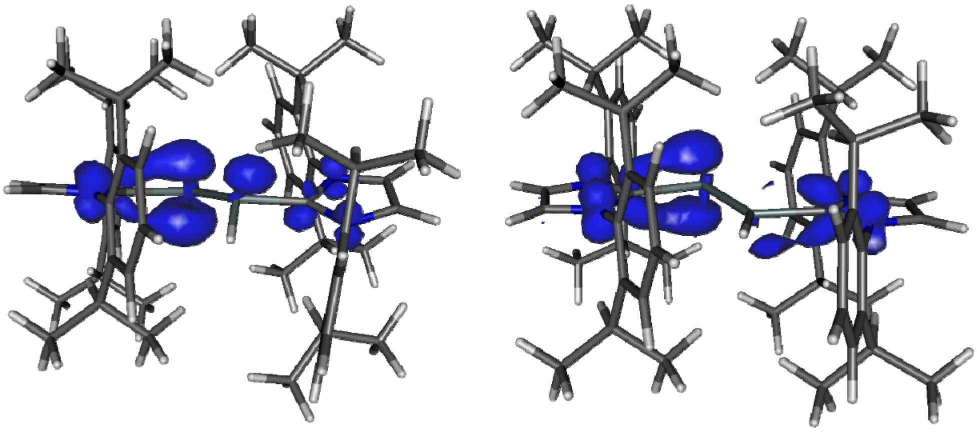

Figure S20. Spin densities of the calculated (B97-D3/RI-JCOSX/def2-TZVP) minimum structures **1H$_{calc}$** (left) and **1H’$_{calc}$** (right).

Table S9: Selected results of the Mulliken spin density analysis of the calculated (B97-D3/RI-JCOSX/def2-TZVP) minimum structures **1H$_{calc}$** and **1H’$_{calc}$**. For numbering of the atoms see Figure S15.

	Si1	C1	N1	N2	H	Si2	C2	N3	N4
**1H$_{calc}$**	0.09	0.10	0.04	0.03	0.00	0.37	0.17	0.05	0.07
**1H’$_{calc}$**	0.06	0.16	0.06	0.05	0.01	0.29	0.18	0.05	0.07

8.4 Comparison of experimental and calculated EPR parameters

Table S10: Comparison of selected calculated (B97-D3/RI-JCOSX/def2-TZVP) EPR parameters of **1H$_{calc}$** and **1H'$_{calc}$** with the experimentally determined values of **1H**. For numbering of the atoms see Figure S15.

	a(Si1) [mT]	*a*(Si2) [mT]	*a*(N1) [mT]	*a*(N2) [mT]	*a*(N3) [mT]	*a*(N4) [mT]	*a*(H) [mT]	g_{iso}
**1H$_{calc}$**	−1.314	−1.811	0.039	0.044	0.078	0.156	0.003	2.00483
**1H'$_{calc}$**	−0.485	−1.380	0.065	0.092	0.093	0.100	−0.482	2.00454
**1H**[a]	0.431	1.725	0.099		0.246		0.607	2.00538

[a]: Experimental data given in *n*-hexane at 298 K. The absolute signs of the hyperfine coupling constants could not be determined experimentally.

8.5 Results of the natural bond order (NBO) calculations

The natural bond orbital (NBO) analysis of **1H$_{calc}$** were carried out at the B3LYP/6-311G**/6-31G* level of theory. For comparison reasons, the NBO analysis of **1H$^{+}_{calc}$** was also carried out. Selected results of the NBO are summarized in Tables S11 and S12. The partial charges obtained by natural population analysis (NPA) are summarized in Table S13.

Table S11: Selected results of the natural bond orbital (NBO) analysis of **1H$^{+}_{calc}$** (B3LYP/6-311G**/6-31G*). For numbering of the atoms see Figure S14.[a]

	occ.	pol. [%]	hyb.	WBI
σ(Si1–Si2)	1.94	61.2 (Si1)<br>38.8 (Si2)	$sp^{1.23}$ (Si1)<br>$sp^{5.59}$ (Si2)	1.6966
π(Si1–Si2)	1.80	60.8 (Si1)<br>39.2 (Si2)	p (Si1)<br>p (Si2)	
σ(Si1–H)	1.98	43.7 (Si1)<br>56.3 (H)	$sp^{2.22}$ (Si1)<br>s (H)	0.9212
σ(Si1–C1)	1.97	25.3 (Si1)<br>74.7 (C1)	$sp^{3.06}$ (Si1)<br>$sp^{1.27}$ (C1)	0.8626
σ(Si2–C2)	1.95	20.7 (Si2)<br>79.3 (C2)	$sp^{7.98}$ (Si2)<br>$sp^{1.25}$ (C2)	0.7392
n(Si2)	1.87		$sp^{0.34}$	

[a]: occ. = occupancy, pol. = polarization, hyb. = hybridization, WBI = Wiberg bond index.

Table S12: Selected results of the natural bond orbital (NBO) analysis of **1H$_{calc}$** (B3LYP/6-311G**/6-31G*). For numbering of the atoms see Figure S14.[a]

	α-spin			β-spin			
	occ.	pol. [%]	hyb.	occ.	pol. [%]	hyb.	WBI
σ(Si1–Si2)	0.97	58.9 (Si1)<br>41.1 (Si2)	$sp^{1.47}$ (Si1)<br>$sp^{6.03}$ (Si2)	0.98	62.9 (Si1)<br>37.1 (Si2)	$sp^{1.28}$ (Si1)<br>$sp^{6.87}$ (Si2)	1.1744
π(Si1–Si2)	–	–	–	0.82	71.7 (Si1)<br>28.4 (Si2)	$sp^{32.05}$ (Si1)<br>$sp^{34.08}$ (Si2)	
σ(Si1–H)	0.99	41.6 (Si1)<br>58.6 (H)	$sp^{2.50}$ (Si1)<br>s (H)	0.99	42.0 (Si1)<br>58.0 (H)	$sp^{2.36}$ (Si1)<br>s (H)	0.8994
σ(Si1–C1)	0.97	27.5 (Si1)<br>72.5 (C1)	$sp^{2.47}$ (Si1)<br>$sp^{1.43}$ (C1)	0.98	24.1 (Si1)<br>75.9 (C1)	$sp^{3.15}$ (Si1)<br>$sp^{1.13}$ (C1)	1.0114
π(Si1–C1)	0.94	56.1 (Si1)<br>43.9 (C1)	$sp^{32.81}$ (Si1)<br>$sp^{15.18}$ (C1)	–	–	–	
σ(Si2–C2)	0.98	21.1 (Si2)<br>78.9 (C2)	$sp^{6.31}$ (Si2)<br>$sp^{1.08}$ (C2)	0.98	21.0 (Si2)<br>79.0 (C2)	$sp^{5.46}$ (Si2)<br>$sp^{1.08}$ (C2)	0.9475
π(Si2–C2)	0.96	52.7 (Si2)<br>47.3 (C2)	p (Si2)<br>p (C2)	–	–	–	
n(Si2)	0.95		$sp^{0.36}$	0.95		$sp^{0.42}$	

[a]: occ. = occupancy, pol. = polarization, hyb. = hybridization, WBI = Wiberg bond index.

Table S13: Comparison of selected partial charges [*e*] obtained by natural population analysis (NPA) of **1H$^{+}_{calc}$** and **1H$_{calc}$** (B3LYP/6-311G**/6-31G*). For numbering of the atoms see Figure S14.

	Si1	Si2	H	C1	C2	Σ(NHC1 ring)[a]	Σ(NHC2 ring)[a]	Σ(NHC1)[b]	Σ(NHC2)[b]
**1H$^{+}_{calc}$**	0.27	0.21	−0.14	0.09	0.07	−0.22	−0.28	0.36	0.30
**1H$_{calc}$**	0.14	0.03	−0.18	0.03	−0.04	−0.45	−0.53	0.05	−0.04

[a]: Σ(NHC1 ring) and Σ(NHC2 ring) are the sums of the partial charges of the heterocyclic ring atoms of the NHC substituents bonded to Si1 and Si2, respectively. [b]: Σ(NHC1) and Σ(NHC2) are the sums of the partial charges of all atoms of the NHC substituents bonded to Si1 and Si2, respectively.

8.6 Results of the TdDFT calculations

The electronic absorption spectrum of **1H** was analyzed by time-dependent density functional theory (TdDFT). The first 75 dipole-allowed electronic excitations of **1H$_{calc}$** were calculated at the B97-D3/RI-JCOSX/def2-TZVP level of theory. The calculated spectrum was simulated by convolution of the oscillator strengths with Gaussian functions applying a value of 1500 cm^{-1} for the full linewidth at half maximum (FWHM), before the spectrum was converted to the nm scale for comparison with the experimental spectrum.

A comparison of the experimental UV-Vis-NIR spectrum of **1H** with the simulated spectrum of **1H$_{calc}$** and a stick plot of the oscillator strengths of the simulated spectrum is depicted in Figure S21. An overview of the calculated energies and assignments of the first electronic transition at 1295 nm and those electronic transitions of **1H$_{calc}$**, which display a relative oscillator strength of >10 % with respect to the electronic transition with the highest oscillator strength at 521 nm, is given in Table S14. Selected α- and β-spin Kohn-Sham orbitals of **1H$_{calc}$** are depicted in Figures S22 and S23.

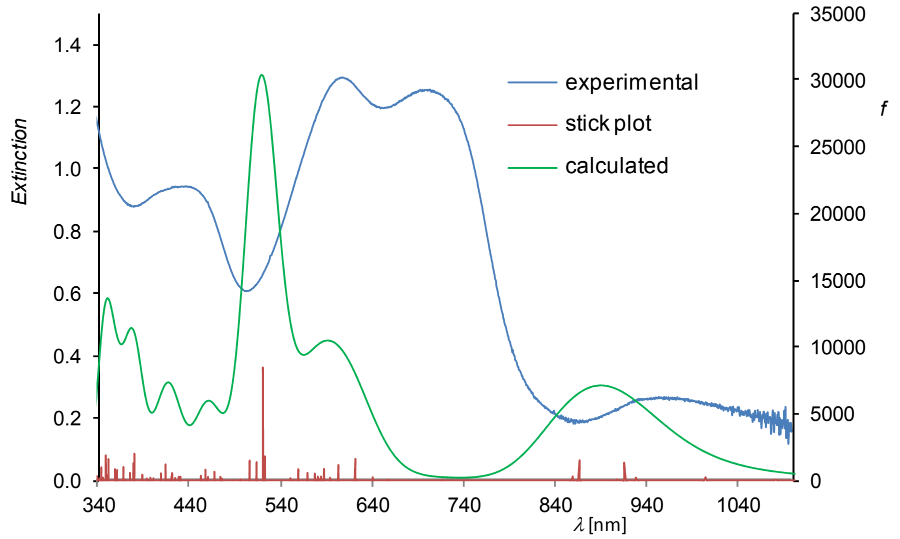

Figure S21. Experimental UV-Vis-NIR spectrum (c = 360 μmol, d = 5 mm, blue curve), calculated spectrum (green curve) and stic plot of the oscillator strengths (red curve) of **1H$_{calc}$**; f is the oscillator strength.

Table S14: Calculated energies and assignments of the electronic transitions of **1H$_{calc}$**.[a]

Excited state	λ [nm]	MO contributions[b]	Contribution [%]	f	f_{rel} [%]
1	1295	HOMO(α)→LUMO(α)	100	0.0004	0.3
5	916	HOMO(α)→LUMO+2(α)<br>HOMO(α)→LUMO+5(α)<br>HOMO(β)→LUMO(β)	27<br>20<br>41	0.0215	15.8
8	868	HOMO(α)→LUMO+5(α)<br>HOMO(α)→LUMO+6(α)<br>HOMO(β)→LUMO(β)	48<br>29<br>11	0.0242	17.8
13	622	HOMO−1(β)→LUMO(β)<br>HOMO(β)→LUMO+3(β)	28<br>54	0.0261	19.1
16	604	HOMO(α)→LUMO+10(α)<br>HOMO(β)→LUMO+3(β)	50<br>11	0.0185	13.6
26	521	HOMO−1(α)→LUMO+2(α)<br>HOMO−1(α)→LUMO+4(α)<br>HOMO(α)→LUMO+8(α)<br>HOMO(α)→LUMO+11(α))	12<br>13<br>13<br>25	0.1361	1.00
28	523	HOMO−1(α)→LUMO+4(α)<br>HOMO−1(α)→LUMO+5(α)	15<br>66	0.0289	21.3
29	514	HOMO−1(α)→LUMO+6(α)	87	0.0219	16.1
30	507	HOMO−1(α)→LUMO+7(α)	88	0.0240	17.6
56	415	HOMO−1(α)→LUMO+9(α)<br>HOMO−1(α)→LUMO+10(α)<br>HOMO−1(α)→LUMO+11(α)	29<br>20<br>27	0.0192	14.1
59	380	HOMO(α)→LUMO+18(α)	47	0.0322	23.7
60	379	HOMO(α)→LUMO+18(α)	49	0.0206	15.1
62	368	HOMO(β)→LUMO+13(β)	47	0.0161	11.9
68	352	HOMO(α)→LUMO+22(α)<br>HOMO(α)→LUMO+22(α)<br>HOMO(α)→LUMO+23(α)	11<br>12<br>27	0.0257	18.9
69	349	HOMO(α)→LUMO+23(α)	68	0.0301	22.1
71	344	HOMO(α)→LUMO+12(α)	73	0.0158	11.6

[a]: λ is the wavelength of the excitation; f is the oscillator strength of the electronic transition; f_{rel} is the relative oscillator strength of the electronic transition with respect to the electronic transition with the highest oscillator strength at 521 nm. [b]: Only MO contributions >10 % are given.

α-Spin orbitals

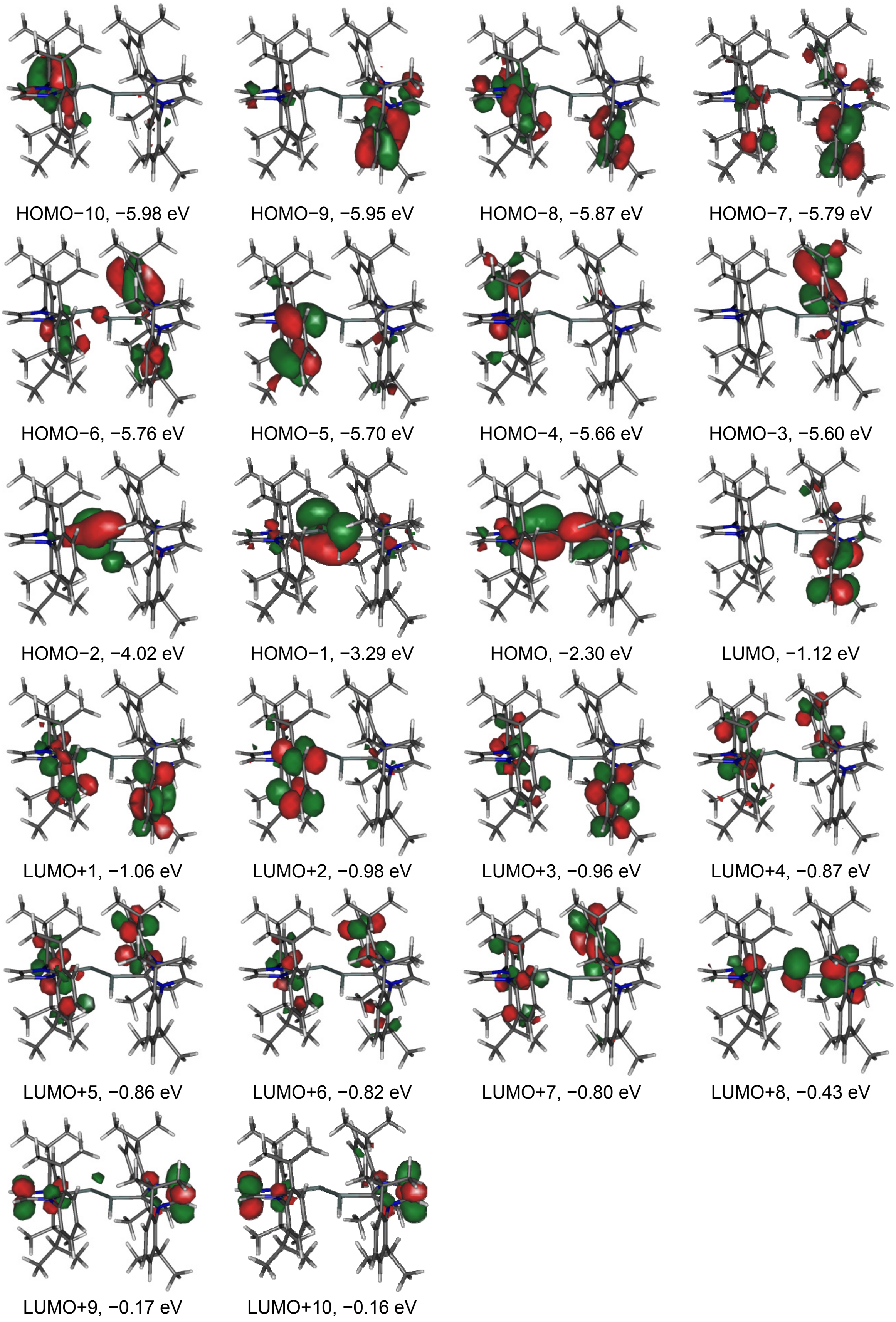

Figure S22. Selected α-spin Kohn-Sham orbitals of **1H$_{\mathbf{calc}}$** and their energy eigenvalues; isosurface value: 0.04 e bohr^{-3}.

β-Spin orbitals

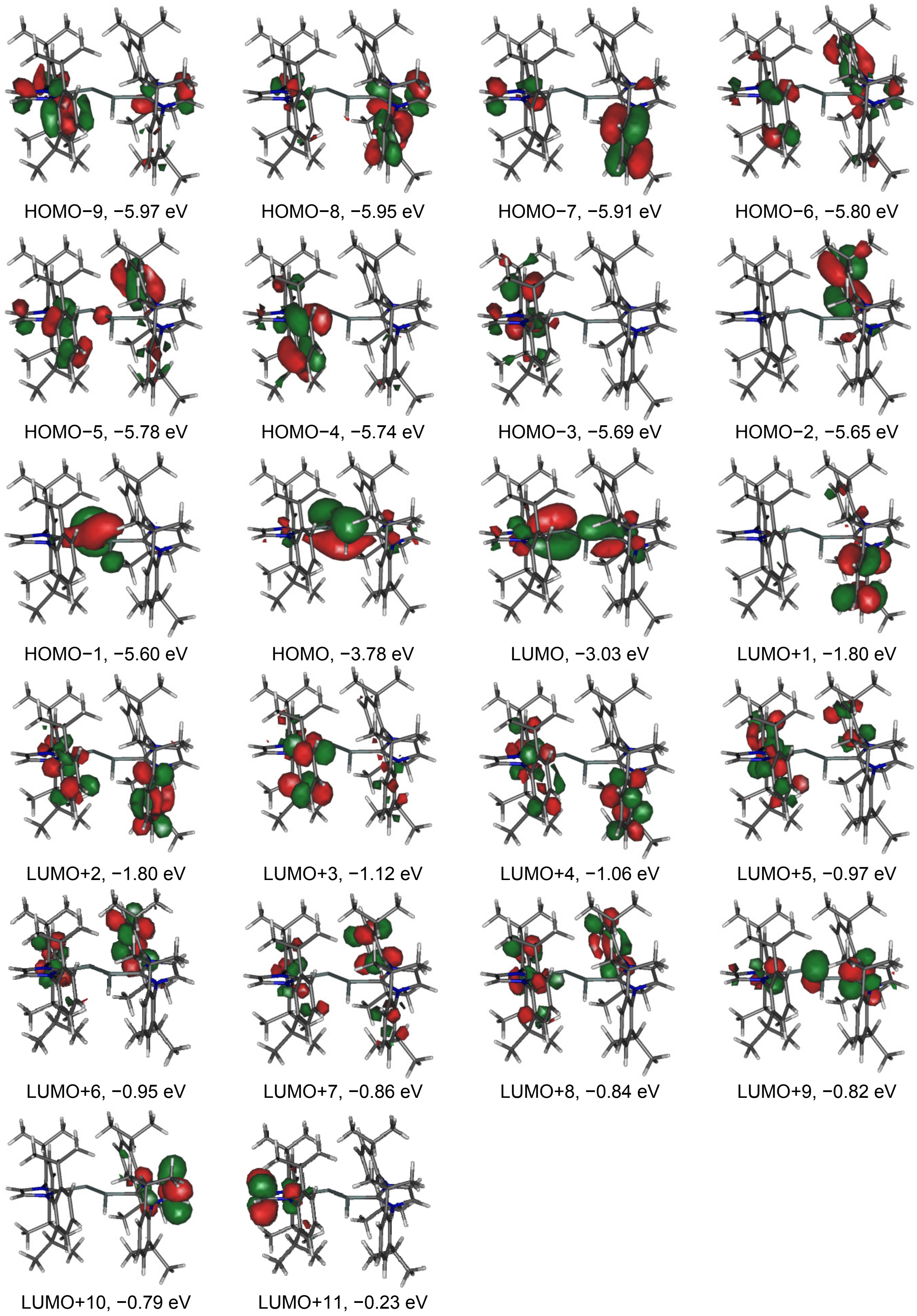

Figure S23. Selected β-spin Kohn-Sham orbitals of $\mathbf{1H_{calc}}$ and their energy eigenvalues; isosurface value: 0.04 e bohr^{-3}.

8.7 Results of the CASSCF calculations

To find out whether a multiconfigurational wavefunction is necessary to describe compound **1H**, second-order Møller-Plesset (MP2) calculations[S18] were performed for **1H$_{calc}$** to determine the relevant orbitals for a complete active space self-consistent field (CASSCF) calculation.[S19] This led to a CASSCF(7,8)/def2-TZVP calculation, which did not converge due to the presence of statically non-correlated orbitals. Successive removal of those orbitals from the active space gave rise to a CASSCF(3,3)/def2-TZVP calculation, which converged. The occupancies of the thereby derived CASSCF(3,3) orbitals (Figure S24) suggest, that static correlation effects can be neglected in the electronic description of **1H$_{calc}$**. The overall wavefunction is described by a major contribution (96 %) from the electronic ground state configuration [2-1-0] and three minor contributions of electronically excited states with the configurations [0-1-2] (3 %), [1-1-1] (0.5 %) and [1-0-2] (0.5%), respectively. These results suggest that DFT methods are sufficient to analyse the electronic structure of compound **1H**.

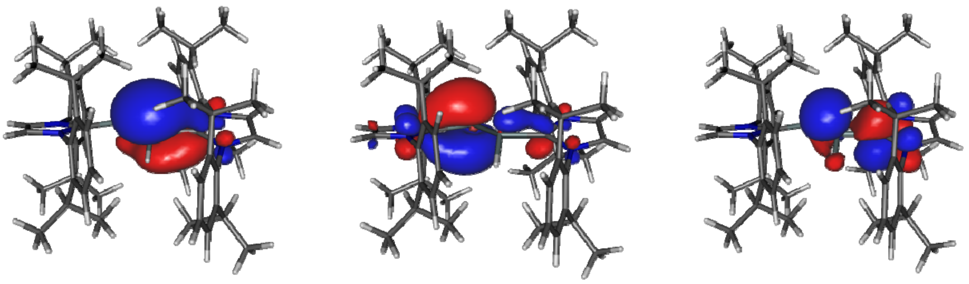

DOMO, π(Si–Si) 1.926 e$^-$, −6.10 eV | SOMO, π*(Si–Si) 0.995 e$^-$, −1.39 eV | LUMO, π*(Si1–C1) 0.080 e$^-$, 5.19 eV

Figure S24. CASSCF(3,3)/def2-TZVP orbitals with their occupancies and energy eigenvalues of **1H$_{calc}$**; iso surface value = 0.04 e bohr^{-3}; DOMO = doubly occupied molecular orbital, SOMO = singly occupied molecular orbital, LUMO = lowest unoccupied molecular orbital.

8.8 Cartesian coordinates [Å] and SCF energies of the calculated structures

$1H^+_{calc}$ (B3LYP/6-311G**/6-31G*)
Energy = −2899.60322742 E_H

Si	-1.190858	-0.283179	0.744687
N	-2.491430	2.215713	-0.391399
N	-3.280963	0.374417	-1.212966
C	-2.270707	0.867969	-0.437485
C	-3.620174	2.547879	-1.127105
H	-3.962835	3.566987	-1.204148
C	-4.112149	1.395625	-1.644525
H	-4.967042	1.202576	-2.272834
C	-1.734498	3.204580	0.354005
C	-2.082960	3.434513	1.701136
C	-1.367901	4.425697	2.385629
H	-1.605656	4.632748	3.424188
C	-0.371533	5.161952	1.752556
H	0.165447	5.930352	2.301726
C	-0.072631	4.931568	0.411841
H	0.691640	5.530960	-0.071886
C	-0.747561	3.950364	-0.324985
C	-3.238521	2.715701	2.394097
H	-3.520357	1.849760	1.786060
C	-4.471161	3.641556	2.478739
H	-4.256787	4.527199	3.087775
H	-5.314384	3.113318	2.938027
H	-4.787593	3.987859	1.488462
C	-2.864317	2.179812	3.787627
H	-1.992514	1.520157	3.739886
H	-3.701267	1.606877	4.202088
H	-2.643579	2.988573	4.492982
C	-0.454160	3.770409	-1.813366
H	-0.809941	2.777606	-2.111605
C	1.046778	3.838318	-2.148655
H	1.447122	4.849620	-2.016588
H	1.204145	3.565444	-3.198003
H	1.635265	3.155807	-1.529310
C	-1.223639	4.816512	-2.650269
H	-2.305582	4.756344	-2.494152
H	-1.029948	4.667691	-3.718666
H	-0.906397	5.832386	-2.388228
C	-3.515605	-1.021078	-1.531623
C	-4.328956	-1.780133	-0.663672
C	-4.579124	-3.109831	-1.024960
H	-5.201861	-3.726214	-0.384117
C	-4.058619	-3.649919	-2.197441
H	-4.273577	-4.681963	-2.460153
C	-3.275868	-2.867846	-3.042926
H	-2.891906	-3.297393	-3.962867
C	-2.985587	-1.533484	-2.733853
C	-4.978819	-1.201127	0.590537
H	-4.538070	-0.217784	0.784280
C	-4.720365	-2.061899	1.841113
H	-5.186542	-3.050272	1.759606
H	-5.147714	-1.572246	2.723487
H	-3.647898	-2.198838	2.012513
C	-6.491385	-0.990786	0.366999
H	-6.685694	-0.335056	-0.489320
H	-6.948668	-0.535932	1.252977
H	-6.999809	-1.943047	0.178235
C	-2.178986	-0.682131	-3.710215
H	-1.887109	0.241782	-3.199385
C	-0.881785	-1.374186	-4.165439
H	-0.261399	-1.660048	-3.309655
H	-0.300500	-0.694454	-4.798505
H	-1.083377	-2.274487	-4.756661

C	-3.040799	-0.287915	-4.928709
H	-3.360707	-1.174703	-5.487729
H	-2.469186	0.351940	-5.610616
H	-3.942425	0.257907	-4.629147
Si	0.762809	0.012569	-0.243010
H	1.256633	0.737380	-1.436521
N	2.365855	-1.951120	1.269096
N	3.585879	-0.689365	-0.011943
C	2.293490	-0.917545	0.378593
C	3.679293	-2.360817	1.423668
H	3.940664	-3.168499	2.088446
C	4.441175	-1.573750	0.623690
H	5.505121	-1.549616	0.450296
C	1.267367	-2.560256	1.993183
C	0.602403	-3.656113	1.405626
C	-0.417517	-4.255516	2.153499
H	-0.952367	-5.104350	1.739530
C	-0.748427	-3.790742	3.424099
H	-1.540361	-4.276138	3.987653
C	-0.058360	-2.718878	3.982963
H	-0.315315	-2.380663	4.982064
C	0.971369	-2.076481	3.284305
C	0.997548	-4.229215	0.047644
H	1.643689	-3.501151	-0.454425
C	1.813017	-5.527740	0.225913
H	1.216261	-6.301969	0.721475
H	2.128454	-5.918108	-0.748395
H	2.711539	-5.363308	0.831399
C	-0.212692	-4.465608	-0.873246
H	-0.801271	-3.552262	-1.000854
H	0.130954	-4.794586	-1.860645
H	-0.876750	-5.245778	-0.484632
C	1.760245	-0.951965	3.950642
H	2.418916	-0.502643	3.199878
C	0.854195	0.173504	4.481818
H	0.191418	-0.178198	5.280403
H	1.466901	0.981899	4.896728
H	0.233775	0.589145	3.681608
C	2.657048	-1.511115	5.075477
H	3.343497	-2.280189	4.703373
H	3.256359	-0.708717	5.520302
H	2.055477	-1.961913	5.872848
C	4.064131	0.364401	-0.887068
C	4.219793	0.083318	-2.259504
C	4.732160	1.107030	-3.065672
H	4.870628	0.930611	-4.127544
C	5.076308	2.344692	-2.528025
H	5.475267	3.122366	-3.173188
C	4.923901	2.584670	-1.165325
H	5.213173	3.548143	-0.756903
C	4.418987	1.599580	-0.306731
C	3.904495	-1.283348	-2.861424
H	3.300558	-1.842917	-2.138657
C	3.080116	-1.184722	-4.157671
H	3.648966	-0.720132	-4.970565
H	2.794114	-2.187431	-4.494212
H	2.166084	-0.602012	-4.006349
C	5.202240	-2.083957	-3.102301
H	5.774658	-2.218949	-2.177784
H	4.970281	-3.076856	-3.503849
H	5.850901	-1.572234	-3.822399
C	4.332203	1.872798	1.193058
H	3.806924	1.037069	1.666793
C	3.530625	3.147716	1.515061
H	2.515583	3.098463	1.108304
H	3.454531	3.275347	2.600794
H	4.015917	4.044489	1.113572
C	5.743513	1.940026	1.814152
H	6.322219	2.770314	1.393874

H	5.676506	2.092265	2.897281
H	6.308533	1.018171	1.635721

1H$_{calc}$ (B3LYP/6-311G**/6-31G*)
Energy = −2899.72620651 E_H

Si	1.199453	0.444432	-0.176644
N	-0.089119	2.159375	-2.413457
N	2.088614	2.190880	-2.246855
C	0.954666	1.608877	-1.666670
C	0.397067	3.027448	-3.387481
H	-0.267485	3.542711	-4.062602
C	1.744794	3.042889	-3.283384
H	2.494129	3.576824	-3.846333
C	-1.499552	1.881830	-2.323780
C	-2.041939	0.878822	-3.153521
C	-3.432362	0.712899	-3.149618
H	-3.880692	-0.051348	-3.777535
C	-4.249144	1.514136	-2.357642
H	-5.326835	1.372824	-2.373145
C	-3.689481	2.496925	-1.544744
H	-4.339215	3.115273	-0.933226
C	-2.305052	2.702686	-1.506167
C	-1.173349	0.021188	-4.069066
H	-0.130414	0.164946	-3.771718
C	-1.309264	0.470344	-5.538671
H	-2.342010	0.360269	-5.891141
H	-0.666167	-0.137633	-6.186465
H	-1.021830	1.519740	-5.667718
C	-1.479182	-1.480577	-3.927275
H	-1.403299	-1.795191	-2.882497
H	-0.765599	-2.065800	-4.519626
H	-2.484857	-1.730131	-4.285860
C	-1.711482	3.818843	-0.651563
H	-0.651063	3.591639	-0.503417
C	-2.351981	3.907753	0.744196
H	-3.394744	4.244774	0.698535
H	-1.804922	4.633025	1.357872
H	-2.321729	2.937849	1.249932
C	-1.809629	5.176719	-1.378853
H	-1.284034	5.161015	-2.339711
H	-1.367432	5.972464	-0.767040
H	-2.856208	5.442193	-1.572400
C	3.458633	1.975554	-1.855921
C	4.199197	0.966805	-2.505002
C	5.554768	0.841826	-2.177001
H	6.152088	0.076003	-2.664088
C	6.149506	1.683835	-1.241629
H	7.204343	1.572084	-1.003621
C	5.395139	2.670582	-0.612034
H	5.869015	3.323666	0.115360
C	4.035953	2.838222	-0.901140
C	3.581546	0.042018	-3.548667
H	2.507513	0.246074	-3.584130
C	3.744892	-1.440816	-3.167179
H	4.799177	-1.742056	-3.142704
H	3.235594	-2.078167	-3.900235
H	3.309947	-1.635052	-2.181886
C	4.155213	0.321085	-4.952509
H	3.997411	1.364147	-5.250286
H	3.672166	-0.320952	-5.698899
H	5.233364	0.123816	-4.989759
C	3.239873	3.944940	-0.216982
H	2.183538	3.792842	-0.456437
C	3.363829	3.888674	1.316799
H	3.056303	2.909642	1.696604
H	2.720327	4.651896	1.771307
H	4.389947	4.084743	1.651227

C	3.649315	5.331508	-0.754314
H	4.705049	5.542866	-0.544626
H	3.049509	6.118774	-0.281778
H	3.504517	5.401490	-1.838605
Si	-0.966928	-0.239807	0.378594
H	-1.925594	-0.878399	-0.576650
N	-0.209559	-1.648646	2.881162
N	-1.955543	-2.527197	1.908404
C	-0.970334	-1.561451	1.722234
C	-0.730013	-2.609461	3.743746
H	-0.270251	-2.810029	4.698121
C	-1.810656	-3.152105	3.143304
H	-2.490786	-3.924927	3.464587
C	0.998522	-0.929271	3.203997
C	0.896202	0.304384	3.877891
C	2.086279	0.931057	4.265913
H	2.043345	1.881582	4.789124
C	3.324208	0.352744	3.998327
H	4.236114	0.855594	4.310050
C	3.397124	-0.867986	3.334532
H	4.368771	-1.307390	3.129766
C	2.239314	-1.536957	2.920986
C	-0.451813	0.925285	4.231406
H	-1.214586	0.444264	3.611836
C	-0.804352	0.660822	5.710007
H	-0.061258	1.111691	6.379104
H	-1.783479	1.090405	5.954824
H	-0.843126	-0.412359	5.929005
C	-0.508336	2.429336	3.914094
H	-0.232307	2.614840	2.871657
H	-1.525698	2.806104	4.071765
H	0.158990	3.013147	4.559495
C	2.347069	-2.893854	2.231253
H	1.364291	-3.136349	1.815577
C	3.343084	-2.880639	1.057433
H	4.373901	-2.727879	1.398513
H	3.311893	-3.842229	0.530714
H	3.095874	-2.084105	0.348879
C	2.711444	-3.998787	3.244843
H	1.972386	-4.069613	4.050915
H	2.762204	-4.974777	2.747004
H	3.688251	-3.805009	3.704251
C	-2.979368	-2.916830	0.974314
C	-4.247559	-2.308822	1.061658
C	-5.246913	-2.763876	0.192318
H	-6.236318	-2.317729	0.238428
C	-4.994201	-3.779584	-0.725511
H	-5.784354	-4.119342	-1.390357
C	-3.732513	-4.365773	-0.789762
H	-3.549814	-5.163862	-1.503754
C	-2.697841	-3.950701	0.057114
C	-4.558082	-1.220920	2.085297
H	-3.612185	-0.904828	2.534986
C	-5.186725	0.026955	1.439491
H	-6.170614	-0.189006	1.005181
H	-5.326558	0.808392	2.196054
H	-4.541795	0.425951	0.650850
C	-5.454255	-1.769270	3.214704
H	-4.986289	-2.621065	3.721529
H	-5.644295	-0.992472	3.965052
H	-6.423315	-2.104652	2.825634
C	-1.339910	-4.644611	0.005342
H	-0.643220	-4.062724	0.615237
C	-0.753881	-4.694872	-1.416448
H	-0.666780	-3.690285	-1.840039
H	0.246939	-5.142135	-1.393230
H	-1.368222	-5.300431	-2.093545
C	-1.425252	-6.059516	0.614668
H	-2.108947	-6.697347	0.041360

H	-0.438552	-6.537939	0.613424
H	-1.785394	-6.029822	1.649263

1H$_{calc}$ (B97-D3/RI-JCOSX/def2-TZVP)
Energy = −2899.073301627194 E_H

Si	7.654838	-6.199433	-0.025952
N	6.388366	-4.661857	-2.359323
N	8.546243	-4.502400	-2.116923
C	7.430271	-5.138075	-1.566646
C	6.850091	-3.766734	-3.320510
H	6.181300	-3.306553	-4.030209
C	8.194306	-3.668587	-3.165039
H	8.930343	-3.087205	-3.697162
C	5.049059	-5.163630	-2.350884
C	4.740468	-6.249427	-3.187775
C	3.420458	-6.714838	-3.181287
H	3.152294	-7.573400	-3.790878
C	2.449594	-6.096506	-2.399463
H	1.429607	-6.471980	-2.408414
C	2.782105	-5.012548	-1.591075
H	2.014807	-4.536748	-0.989313
C	4.096281	-4.539772	-1.526776
C	5.795862	-6.919171	-4.051809
H	6.709680	-6.322819	-3.986846
C	5.383393	-6.956599	-5.531283
H	4.499609	-7.585548	-5.685298
H	6.196195	-7.368297	-6.140517
H	5.151486	-5.952319	-5.902853
C	6.137056	-8.320293	-3.522643
H	6.499753	-8.261023	-2.492887
H	6.915248	-8.782052	-4.142635
H	5.257651	-8.973573	-3.534769
C	4.486426	-3.380091	-0.625791
H	5.558349	-3.481120	-0.423057
C	3.770562	-3.413460	0.729555
H	2.696590	-3.216844	0.632248
H	4.188617	-2.644069	1.388119
H	3.907394	-4.388714	1.208755
C	4.260448	-2.035060	-1.339310
H	4.830288	-1.979083	-2.271173
H	4.573886	-1.203070	-0.697223
H	3.199075	-1.899143	-1.578976
C	9.888206	-4.713330	-1.662476
C	10.610940	-5.799981	-2.190362
C	11.934992	-5.957482	-1.772114
H	12.524985	-6.781796	-2.160544
C	12.505213	-5.076229	-0.853875
H	13.536652	-5.216255	-0.539534
C	11.756997	-4.028121	-0.327787
H	12.206513	-3.360494	0.402399
C	10.426879	-3.828408	-0.715826
C	9.969630	-6.754121	-3.184419
H	8.891991	-6.726332	-2.999766
C	10.414514	-8.208944	-2.991410
H	11.464695	-8.357156	-3.269126
H	9.811314	-8.868605	-3.624021
H	10.285411	-8.521436	-1.951608
C	10.212388	-6.292917	-4.632092
H	9.831884	-5.280013	-4.799428
H	9.707073	-6.963969	-5.336485
H	11.283969	-6.294821	-4.863826
C	9.598696	-2.713988	-0.101074
H	8.577534	-2.811450	-0.482166
C	9.519051	-2.864763	1.425458
H	9.141193	-3.857835	1.683052
H	8.839809	-2.114118	1.846497
H	10.500810	-2.733736	1.896025

C	10.122833	-1.329256	-0.514004
H	11.143140	-1.167384	-0.146814
H	9.486274	-0.539895	-0.098131
H	10.137686	-1.221686	-1.604571
Si	5.516655	-6.982091	0.352548
H	4.519660	-7.591122	-0.579631
N	6.289169	-8.343091	2.844997
N	4.449627	-9.144619	1.991794
C	5.469324	-8.231457	1.730735
C	5.795884	-9.286446	3.738734
H	6.313520	-9.511798	4.656934
C	4.654345	-9.787208	3.208758
H	3.970648	-10.537457	3.571127
C	7.555711	-7.692051	3.006236
C	7.588400	-6.403547	3.565243
C	8.842171	-5.815646	3.755455
H	8.907010	-4.822084	4.186830
C	10.006456	-6.474521	3.370418
H	10.970883	-5.991571	3.506083
C	9.940605	-7.734935	2.785735
H	10.854239	-8.224518	2.461270
C	8.710924	-8.371428	2.587837
C	6.311689	-5.681330	3.959774
H	5.485183	-6.166663	3.430363
C	6.053194	-5.818771	5.469633
H	6.855683	-5.345178	6.047682
H	5.106931	-5.337019	5.744750
H	5.999640	-6.871695	5.766684
C	6.314317	-4.211873	3.519282
H	6.509229	-4.138505	2.444676
H	5.338474	-3.758155	3.726849
H	7.071238	-3.626044	4.051787
C	8.643531	-9.737290	1.924784
H	7.589535	-9.976368	1.756352
C	9.320410	-9.726848	0.546706
H	10.396211	-9.534572	0.627817
H	9.188492	-10.695588	0.049632
H	8.879470	-8.944464	-0.079149
C	9.227192	-10.826447	2.839738
H	8.701970	-10.857063	3.800228
H	9.140135	-11.812987	2.368098
H	10.288420	-10.641788	3.042690
C	3.383449	-9.435528	1.081572
C	2.237274	-8.620312	1.108760
C	1.241342	-8.877431	0.162607
H	0.345277	-8.264680	0.142337
C	1.388950	-9.904383	-0.768102
H	0.608282	-10.081506	-1.504585
C	2.523788	-10.709877	-0.756545
H	2.617396	-11.511539	-1.483556
C	3.546813	-10.496699	0.174805
C	2.093001	-7.527814	2.155817
H	3.101528	-7.157703	2.374007
C	1.267256	-6.330065	1.676976
H	0.205019	-6.581897	1.573110
H	1.339879	-5.512770	2.402933
H	1.634227	-5.965839	0.715807
C	1.501584	-8.101834	3.457643
H	2.113761	-8.917826	3.852976
H	1.439336	-7.321113	4.224973
H	0.491042	-8.488618	3.280470
C	4.798846	-11.359739	0.180905
H	5.285919	-11.226918	1.152460
C	5.795456	-10.893673	-0.892632
H	6.081729	-9.851279	-0.731460
H	6.700961	-11.510127	-0.866447
H	5.353256	-10.975409	-1.891844
C	4.482984	-12.855451	0.037583
H	4.094657	-13.097376	-0.957982

H	5.395230	-13.444813	0.181713
H	3.742109	-13.179581	0.777065

1H’$_{calc}$ (B97-D3/RI-JCOSX/def2-TZVP)
Energy = −2899.071227022923 E_H

Si	7.24284079667758	-6.25759931729236	-0.15224872866755
N	6.56785820632848	-4.90435577141306	-2.66043268322727
N	8.63483037991219	-4.53754749677159	-2.04236692603339
C	7.47993878892924	-5.22821439160652	-1.66023894833314
C	7.13324303226881	-4.04178286063014	-3.59247680500000
H	6.56974460770708	-3.68928979907850	-4.44121487459585
C	8.41003261722604	-3.80694697732009	-3.20674143499767
H	9.18031326625249	-3.18909341353486	-3.63824387965522
C	5.19624358424609	-5.31081822176705	-2.67427409918952
C	4.83388738782835	-6.44086250787783	-3.42306178012880
C	3.48344495793786	-6.80627132597069	-3.42738425234940
H	3.16901433805843	-7.68732594052281	-3.97953646681351
C	2.54289545628570	-6.07104785762583	-2.71379852335084
H	1.49964530703393	-6.37738386311601	-2.72013056588592
C	2.93277207548839	-4.95827280773812	-1.97258707204375
H	2.18933803655296	-4.40032451569761	-1.41174282457253
C	4.27202732945944	-4.56350718854963	-1.92171232092613
C	5.86333264537370	-7.25398528862655	-4.18789925914624
H	6.84594074072010	-6.81470193566439	-3.98951572677722
C	5.61815414804214	-7.17744265255827	-5.70311371944866
H	4.64968513780582	-7.61772860037698	-5.96734185107652
H	6.39647722416001	-7.72471421960759	-6.24710822544377
H	5.61962611875700	-6.13832977599925	-6.05160924827623
C	5.91303823569734	-8.70716679733646	-3.69403657740429
H	6.09034148898321	-8.73100488867555	-2.61418580631602
H	6.71907918949137	-9.25217317269684	-4.19917256471793
H	4.97305926324071	-9.23458371790065	-3.89498040612507
C	4.71254679568822	-3.36422203387089	-1.09964399541824
H	5.79432459659870	-3.44405687090341	-0.96209130877717
C	4.09550808831894	-3.35480973091924	0.30458916213341
H	3.01476023827150	-3.17577783793008	0.27485718062570
H	4.54873984455204	-2.55695523248560	0.90369373648133
H	4.27671082015067	-4.31073683213512	0.80594099327836
C	4.43148800031326	-2.05186857452690	-1.85132765029848
H	4.93018686074739	-2.04212821989335	-2.82536348756346
H	4.79405078315680	-1.19299597189591	-1.27323996892792
H	3.35597006411880	-1.92055443498368	-2.01837522976326
C	9.79372307585192	-4.39825478761720	-1.21590898668182
C	10.79731775271876	-5.38170593645867	-1.28023061640072
C	11.90566261098352	-5.22639108695261	-0.44371072576300
H	12.69910662603874	-5.96712855784844	-0.45992200377493
C	12.00150422108925	-4.14013188458175	0.42559462404166
H	12.86913453377272	-4.04301285074961	1.07419033587723
C	10.99358154229108	-3.18398574410339	0.46854712984233
H	11.07828293384926	-2.34152283958516	1.15012859251786
C	9.86419290908491	-3.29352618897629	-0.35251203280886
C	10.66729815004146	-6.55070086022082	-2.24306535687051
H	9.60614594364779	-6.82967714907320	-2.25971105945763
C	11.46183494873901	-7.78601564641451	-1.81021982334873
H	12.54306031020305	-7.62763842503403	-1.90635236450606
H	11.19743250199187	-8.63876131022989	-2.44434797005555
H	11.24129422348666	-8.05059387712619	-0.77434653721464
C	11.07109048716098	-6.13622370808873	-3.67087302804850
H	10.45891073283519	-5.31018318383310	-4.04238689870360
H	10.95100028019503	-6.98100454796317	-4.35965841434956
H	12.12184356575520	-5.82333718020028	-3.69363124991325
C	8.77784122701269	-2.23149660121061	-0.31456758400957
H	7.96970744475487	-2.55321265476060	-0.97638408150987
C	8.16875570061137	-2.08019501185120	1.08578286221205
H	7.72766588898049	-3.02477904446839	1.40964227356356
H	7.38126907229787	-1.31708305220432	1.07590144053606
H	8.92036067403308	-1.77965426110847	1.82461237590621

C	9.30680212036382	-0.89000977000075	-0.85029119021336
H	10.11539834791870	-0.50392646091462	-0.21843267947747
H	8.50437704126491	-0.14364134161723	-0.86946362667228
H	9.69911771258149	-0.99843202473424	-1.86727464050174
Si	5.25695975788060	-7.38344500347915	0.01609088280842
N	6.39092698217206	-8.48796651268054	2.64847375120460
N	4.35465289068701	-9.06117583273656	2.12565895316225
C	5.43365740230146	-8.30468711962809	1.65260290604657
C	5.90970883736586	-9.29814521446793	3.67362393657312
H	6.51400637510049	-9.53670873816113	4.53424473935947
C	4.64377882836254	-9.65468752690905	3.34347556221222
H	3.92592588552627	-10.27795487940381	3.85250185613879
C	7.74402540557555	-8.03514675608683	2.58489294186375
C	8.11284623458814	-6.89620772702242	3.31775252559167
C	9.45432443472155	-6.49979344705967	3.26759907806709
H	9.76961197608749	-5.60871077164544	3.80298053379345
C	10.38465800255247	-7.22078002600352	2.52649553995566
H	11.41990963248868	-6.89195928655123	2.49154421751249
C	9.99068508144474	-8.34909598549486	1.81042268760489
H	10.72762878657600	-8.90320204208381	1.23794371319584
C	8.65918883679456	-8.77210018626613	1.80810898004214
C	7.09895074311203	-6.10467783128377	4.12532030639053
H	6.12373669355930	-6.58336937079350	3.99533273516688
C	7.43337068003091	-6.13408376011535	5.62504397078002
H	8.39792209455846	-5.65280637590128	5.82446904848165
H	6.66668536068956	-5.60235197886619	6.19992158461649
H	7.49144576835729	-7.16261505475294	5.99844191788823
C	6.97018264577916	-4.66739528333616	3.60278444287297
H	6.70400217579092	-4.67854955130757	2.54124081447917
H	6.19113638348105	-4.12752332486649	4.15308157990218
H	7.90967509676203	-4.11460889629902	3.71645288211944
C	8.21652314180758	-9.99906756649595	1.02889163369287
H	7.13606449602101	-9.91729949147301	0.88599452670458
C	8.82985800198394	-10.07101637734751	-0.37383892914016
H	9.91112117607412	-10.24491900415584	-0.34172569122540
H	8.37786309553574	-10.89796154423941	-0.93266547580175
H	8.63811305835808	-9.14374245347611	-0.92242516102436
C	8.49230024513285	-11.28246096958846	1.83104932630395
H	7.99730685709540	-11.25246089518629	2.80640150356925
H	8.12143382007077	-12.16025112847452	1.28845369740021
H	9.56801220414695	-11.41366373617432	1.99807365493587
C	3.18205516866119	-9.33757896295845	1.35104388270920
C	2.09296374734763	-8.45348151196517	1.42513204736161
C	0.95842952065884	-8.76079259277206	0.66742289637536
H	0.09575062637360	-8.10175952121634	0.70302912448450
C	0.92110779399643	-9.89696142565525	-0.13803242478953
H	0.03030959873962	-10.11687434993044	-0.72171734538270
C	2.01902355978118	-10.75006846233872	-0.20039279292054
H	1.98068266816251	-11.63163795372959	-0.83476179349325
C	3.17643919366299	-10.48560670556063	0.54032473583814
C	2.13732686874112	-7.22596945453531	2.31685041728311
H	3.19015088016847	-7.02217315906465	2.53611386033588
C	1.57609787890467	-5.97963251217427	1.62127343365851
H	0.49356122688557	-6.05011522371393	1.46652757662430
H	1.76537236025599	-5.09076085431621	2.23397168301534
H	2.06026884453763	-5.83872461661843	0.65137942218163
C	1.42043040999954	-7.50207230149995	3.65018779154208
H	1.87390406683970	-8.35244643015086	4.17129912290890
H	1.47784280747041	-6.62660717476665	4.30811212735339
H	0.36172398219008	-7.73413326528185	3.48281601502633
C	4.37693258603583	-11.41192162342030	0.45057284006164
H	5.15956405065947	-11.01098658525102	1.09965365946088
C	4.94698211286188	-11.44630744996560	-0.97575044162508
H	5.19203268839233	-10.43454355893215	-1.30807959418817
H	5.85759722050403	-12.05527778270178	-1.00712025409520
H	4.22737018333511	-11.87649581436818	-1.68214497783616
C	4.03814355864529	-12.82158602674468	0.96122747859494
H	3.27172395692488	-13.29532904403956	0.33696433308989
H	4.92983354863312	-13.45829280796384	0.94295576167323

H	3.66062400016499	-12.78714578856175	1.98912400960849
H	8.57393076995685	-6.14934735656170	0.50576060641518

9. References

[S1] M. I. Arz, M. Straßmann, D. Geiß, G. Schnakenburg, A. C. Filippou, *J. Am. Chem. Soc.* **2016**, *138*, 4589.

[S2] a) K. Fredenhagen, G. Cadenbach, *Z. Anorg. Allg. Chem.* **1926**, *158*, 249; b) W. Rüdorff, E. Schulze, *Z. Anorg. Allg. Chem.* **1954**, *277*, 156.

[S3] I. Chávez, A. Alvarez-Carena, E. Molins, A. Roig, W. Maniukiewicz, A. Arancibia, V. Arancibia, H. Brand, J. M. Manríquez, *J. Organomet. Chem.* **2000**, *601*, 126.

[S4] C. H. Hamann, W. Vielstich, *Elektrochemie*, 3., vollständig überarbeitete Auflage, Wiley-VCH, Weinheim, **1998**.

[S5] G. A. Bain, J. F. Berry, *J. Chem. Educ.* **2008**, *85*, 532.

[S6] H. Lueken, *Magnetochemie*, Teubner Studienbücher, Stuttgart, **1999**.

[S7] SADABS, *2009/2*, AXS, 2009.

[S8] G. M. Sheldrick, *SHELXS97* and *SHELXL97*, University of Göttingen, Germany, **1997**.

[S9] S. Stoll, A. Schweiger, *J. Magn. Reson.* **2006**, *178*, 42.

[S10] a) C. Lee, W. Yang, R. G. Parr, *Phys. Rev. B* **1988**, *37*, 785; b) A. D. Becke, *J. Chem. Phys.* **1993**, *98*, 5648.

[S11] P. C. Hariharan, J. A. Pople, *Theor. Chim. Acta* **1973**, *28*, 213.

[S12] a) S. Grimme, S. Ehrlich, L. Goerigk, *J. Comp. Chem.* ***2011***, *32*, 1456; b) S. Grimme, J. Antony, S. Ehrlich, H. Krieg, *J. Chem. Phys*. **2010**, *132*, 154104.

[S13] a) F. Neese, *J. Comput. Chem.* **2003**, *24*, 1740; b) F. Neese, F. Wennmohs, A. Hansen, U. Becker, *Chem. Phys.* **2009**, *356*, 98.

[S14] a) A. Schäfer, H. Horn, R. Ahlrichs, R. *J. Chem. Phys*. **1992**, *97*, 2571; b) F. Weigend, R. Ahlrichs, *Phys. Chem. Chem. Phys.* **2005**, *7*, 3297.

[S15] F. Neese, *WIREs Comput. Mol. Sci*. **2012**, *2*, 73.

[S16] R. S. Mulliken, *J. Chem. Phys.* **1995**, *23*, 1833.

[S17] NBO Version 3.1, E. D. Glendening, A. E. Reed, J. E. Carpenter, F. Weinhold.

[S18] M. Head-Gordon, J. A. Pople, M. J. Frisch, *Chem. Phys. Lett.* **1988**, *153*, 503.

[S19] B. O. Roos, P. R. Taylor, P. E. M. Siegbahn, *Chem. Phys.* **1980**, *48*, 157.
